# Quantum Cardiovascular Medicine: From Hype to Hope—A Critical Review of Real-World Applications

**DOI:** 10.3390/jcm14176029

**Published:** 2025-08-26

**Authors:** Marek Tomala, Maciej Kłaczyński

**Affiliations:** 1 Center for Invasive Cardiology, Electrotherapy, and Angiology, 33-300 Nowy Sacz, Poland; 2 Clinical Research Center Intercard, 30-514 Krakow, Poland; 3 Department of Mechanics and Vibroacoustics, Faculty of Mechanical Engineering and Robotics, AGH University of Krakow, Al. Mickiewicza 30, 30-059 Krakow, Poland; maciej.klaczynski@agh.edu.pl

**Keywords:** quantum medicine, cardiovascular diagnostics, magnetocardiography, quantum machine learning, SQUID technology, quantum sensors, cardiac imaging, precision medicine

## Abstract

**Context:** As quantum technologies advance with innovations in cardiovascular medicine, it can be challenging to distinguish genuine clinical progress from mere ideas. There will also be difficult transitions involved in moving technology from proof of concept demonstrated in the lab. This transition is complicated by the excitement and hype that comes with any new technology. **Aim:** This work aims to assess what quantum technologies are available in cardiovascular medicine for real-world use, to identify which applications are closer to clinically relevant translation, and to differentiate realistic advances from advanced-not-yet realities. **Methods:** A narrative review was conducted using PubMed, EMBASE, Scopus, Web of Science, IEEE Xplore, and arXiv. While real-world use of technologies was prioritized, we included all theoretical literature, regardless of date of publication. Search terms were a combination of the vocabulary of quantum technologies and the vocabulary of cardiovascular medicine. Peer-reviewed publications included primary research, reviews, theoretical works, and conference proceedings. Two reviewers independently screened all citations, and any disagreements were resolved through consensus discussion. **Results:** We identified three core application areas: (1) quantum sensing, such as cardiac magnetometry, where there is potential for SQUID magnetocardiography to be used for detecting cardiomyopathy; (2) quantum computing for cardiovascular risk prediction, and (3) next-generation quantum sensors for mobile cardiac imaging. **Conclusions:** Quantum technology in cardiovascular medicine represents modest promise in select applications, most notably, magnetocardiography. To go from “hype to hope”, clinical trials will be required to identify application domains where quantum technologies outweigh the challenges of implementation in clinical practice.

## 1. Introduction

Cardiovascular diseases (CVDs) represent the leading cause of death, with 19.8 million deaths in 2022. CVDs account for about 32% of all deaths globally [1]. In 2020, more than 523 million people were living with ischemic heart disease, so there is a long way to go to improve the identification, prediction, and treatment of CVDs early on in their course [2]. Quantum technology offers a new paradigm of solutions through fundamentally different processes and mechanisms that are not merely faster computationally but offer new capabilities in representation, sensing, and learning from data [3]. Quantum machine learning algorithms—such as quantum-enhanced support vector machines or kernel methods—can perform explicit analysis of high-dimensional datasets in a logarithmic time while finding latent patterns among sparse clinical features or genomic profiles with diminished training burden [3,4]. Quantum sensors—which include nitrogen-vacancy centers in diamonds and superconducting quantum interference devices (SQUIDs)—are particularly sensitive, detecting biomagnetic cardiac signals at femtotesla levels. These sensors can revolutionize magnetocardiography (MCG), as they do not require cryogenics and shielding [5]. Proof-of-concept quantum sensors have demonstrated detection of stable cardiac fields in ambient conditions, providing non-contact, real-time electrophysiological mapping. At this stage, hybrid quantum-classical models are being explored to augment hemodynamic simulations using a digital twin approach and offer a rapid molecular-level approach to modeling lipid interactions and plaque destabilization. Encouraged applications include piloting a cloud-accessible quantum machine learning (QML) pipeline for risk prediction of atrial fibrillation (AF) and potentially quantum-enhanced reconstruction of coronary computed tomography angiography (CCTA).

The fusion of quantum computation with cardiovascular diagnostics provides not just a shift in scale, but a change in nature, comprising a wide range of capabilities such as the quantum state encoding of patient information, superposition-based evaluations of hypotheses, or the entanglement-based correlation modeling of various states. Each mechanism builds a framework for discovering clinically meaningful patterns and phenotypes that classical systems might miss, holding monumental importance for diagnostics, device development, and precision prevention.

## 2. Methods

### 2.1. Strategy for Literature Search

#### 2.1.1. Database Selection

We conducted extensive searches across multiple databases that cover both medical and physics literature. The medical databases we searched were PubMed/MEDLINE (U.S. National Library of Medicine, Bethesda, MD, USA), EMBASE (Elsevier, Amsterdam, Netherlands), and Scopus (Elsevier, Amsterdam, The Netherlands). The databases covering physics and engineering fields were Web of Science (Clarivate Plc, London, UK), IEEE Xplore (Institute of Electrical and Electronics Engineers [IEEE], Piscataway, NJ, USA), and arXiv (Cornell University, Ithaca, NY, USA). We also utilized additional search tools such as Google Scholar (Google LLC, Mountain View, CA, USA), PubMed Central (U.S. National Library of Medicine, Bethesda, MD, USA), and the Cochrane Library (John Wiley & Sons, Hoboken, NJ, USA).

#### 2.1.2. Search Terms and Strategy

Search strategies were developed iteratively and through consultation with experts—“One strategy combined controlled vocabulary—MeSH (U.S. National Library of Medicine, Bethesda, MD, USA) and Emtree (Elsevier, Amsterdam, Netherlands)—with free-text terms. Terms pertinent to the quantum-related concepts included quantum computers, quantum sensors, quantum imaging, quantum mechanics, quantum coherence, quantum entanglement, quantum dots, quantum biology, nitrogen vacancy centers, and diamond magnetometers. Terms relevant to cardiovascular treatment included cardiovascular, cardiac, cardiology, heart disease, coronary artery disease, arrhythmia, heart failure, magnetocardiography, cardiac imaging, and myocardial infarction. Boolean operators were used to connect and group concepts according to the syntax of the respective database.

#### 2.1.3. Search Parameters

The primary search was for contemporary applications (1 January 2015 to 30 June 2025), with seminal theoretical works and foundational studies included, regardless of publication date. We adopted this temporal approach because it is crucial to recognize that, in systematic literature searches over the last decade, the goal was to identify current clinical applications of advances in quantum theory and technology. These seminal quantum physics papers and early biomedical work involving quantum themes established the theoretical underpinnings, which are necessary for understanding and examining contemporary developments in quantum cardiovascular medicine. There were no language restrictions; however, if the studies were published in a language other than English, we had to have available abstracts in English. We accepted technical reports, published articles, pre-prints, and conference proceedings as relevant publications. The initial searches were conducted from January 2025 to 30 June 2025.

### 2.2. Study Selection Process

#### 2.2.1. Screening Procedures

Two authors independently performed a two-stage screening, including title/abstract screening followed by a full-text screening. Discrepancies were resolved by discussion, and a third reviewer was used to settle any remaining disagreements.

#### 2.2.2. Inclusion Criteria

We defined eligibility criteria before conducting our review, and we provide our eligibility criteria in Table 1. Studies were eligible if they applied conceptual or methodological principles of quantum science to the field of cardiovascular medicine at any level, theoretical, preclinical, or clinical.

#### 2.2.3. Exclusion Criteria

Exclusions included applications of classical physics without any quantum component, quantum studies with no cardiovascular applications, studies that were not available in full text, editorial opinions that did not give any data or support for their opinion, and duplicates of the same research.

#### 2.2.4. Selection Results

The search retrieved a total of 634 records, including PubMed: 182, EMBASE: 141, Scopus: 161, Web of Science: 57, IEEE Xplore: 49, arXiv: 26, and grey (convention) literature: 18. We removed 112 duplicates and 32 automatically excluded records, leaving 490 records for title/abstract screening. Four hundred thirty documents were excluded (primarily due to the absence of a cardiovascular or actual quantum component).

**Figure 1:**
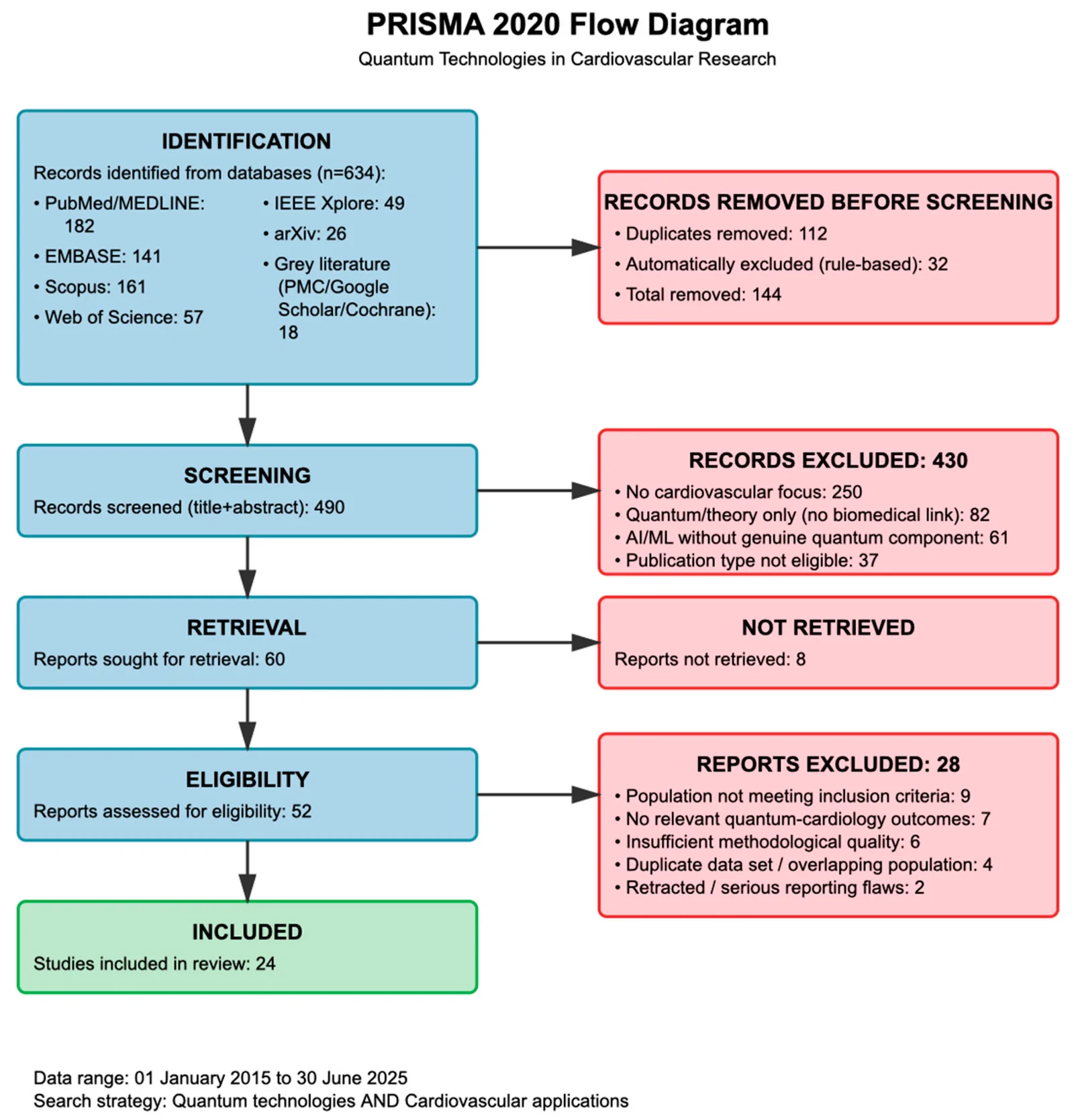
Preferred Reporting Items for Systematic Reviews and Meta-Analyses (PRISMA) flow diagram delineating the process of article selection and review for this study on quantum computing in cardiology. Initially, 634 articles were identified through database searches. After the removal of duplicates, 234 unique articles were screened based on their titles and abstracts. Subsequently, full-text reviews were conducted on the remaining 52 articles to evaluate their content and quality. Ultimately, 24 articles were selected for an in-depth review of their relevance and quality.

After this screening, we aimed to obtain the full text for 60 articles; however, we were unable to obtain 8 of them. A total of 52 full texts were thoroughly reviewed, and at this point, 28 were excluded (see summary Table 1). Overall, we included 24 studies for qualitative synthesis in the review. The final study selection process is presented in Figure 1 (PRISMA 2020 flow diagram [6]).

### 2.3. Data Extraction and Quality Assessment

#### 2.3.1. Data Extraction Framework

Data extraction forms included a standard form for study characteristics including authors, year of publication, and study type; a form for identifying two components for quantum technology; a coding sheet for the cardiovascular applications that stated clinical indications and patient populations; a form for summarizing results and performance measures against usual care; a form for identifying if the study had progressed from theoretical to clinical application; and a form to review mentioned limitations.

#### 2.3.2. Quality Assessment

A multi-dimensional framework identified the unique difficulties of evaluating interdisciplinary quantum–medical research. Clinical studies were rated using a modified Newcastle–Ottawa Scale, technical studies were rated for technical strength, experimental rigor, and reproducibility, and theoretical papers were rated for theoretical rigor, biological plausibility, and testability of predictions. Possible interdisciplinary integration (1–5 scale) was rated for overall success at integrating quantum physics and cardiovascular medicine [7].

### 2.4. Data Synthesis and Analysis

#### Synthesis Approach

The syntheses were narrative and were grouped by application domain (diagnostic, therapeutics, fundamental research), type of quantum technology (sensing, computing, imaging), and stage of the clinical translation (theoretical, proof of concept, preclinical, clinical). This structure helped organize the distribution and thus identify patterns, gaps, and opportunities in the field of quantum cardiovascular research.

### 2.5. Limitations and Bias Mitigation

#### 2.5.1. Recognized Limitations

Quantum technology continues to change rapidly; this review represents a static undertaking. The lack of data to support long-term clinical outcome statements limits our conclusions about the ultimate clinical relevance of quantum technologies. Positive publication bias may lead us to over appreciate the current capabilities of quantum technology. Quality judgments among disciplines are almost impossible, given that there are no universal standards for establishing quality in publications. Language bias was possible; however, some literature not available in English was included (with English abstract). The absence of registering a prospective protocol was neither feasible nor usual for this type of narrative review, with limited transparency concerning possible post hoc analytical decisions.

#### 2.5.2. Bias Reduction

We took some steps to reduce bias in our process by searching the grey literature and including conference proceedings and preprints. Screening of records was performed by two reviewers independently, from two different disciplinary perspectives. We used explicit eligibility criteria and quality appraisal processes that were specific to our research question. The search was also updated for currency up to the point of writing the manuscript.

#### 2.5.3. PRISMA 2020 Caveats

While this is a narrative review, we incorporated some elements of PRISMA 2020 to help promote greater transparency. We have reported a detailed search strategy, an inclusion/exclusion table, and a PRISMA-style flowchart (Figure 1) to give a summary of the process.

PRISMA: elements that do not apply to a narrative review of evidence synthesis, such as protocol registration or formal risk-of-bias assessments, have not been included, as they were not intended for the methodological scope of this review.

## 3. Quantum Sensing Technologies in Cardiovascular Medicine

### 3.1. Fundamental Principles and Technological Platforms

Quantum sensing represents an advanced approach in measurement that leverages fundamental principles of quantum mechanics to measure physical quantities with exceptional sensitivity and spatial resolution. Superconducting Quantum Interference Devices (SQUIDs) are based on two basic principles of quantum mechanics: magnetic flux quantization in a superconducting loop and the Josephson effect. A magnetic flux is quantized in a superconducting loop, which is reported in units of the quantum of magnetic flux (Φ_0_), which is defined as Φ_0_ = h/2e ≈ 2.07 × 10^−15^ Wb. Therefore, as the SQUID detects the magnetic field, the magnetic flux begins to induce a current, which in turn causes a voltage that is periodic in the applied flux. This means that a single SQUID can represent unbelievably sensitive measurements for magnetic fields [8].

**Figure 2:**
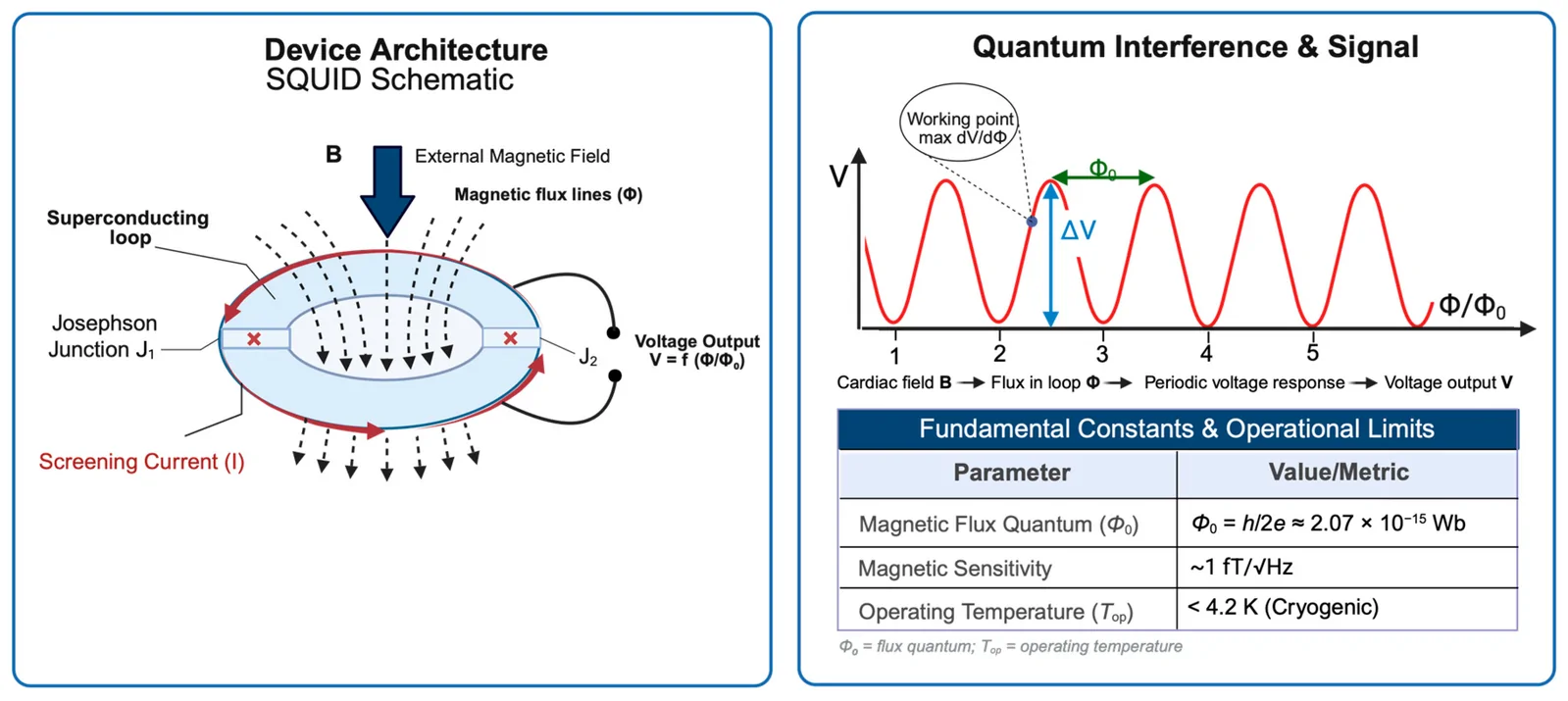
The structure of a DC SQUID (Superconducting Quantum Interference Device) magnetometer for biomedical applications and the quantum interference signal it produces. The **left** panel shows the superconducting loop that contains two Josephson junctions (J_1_ and J_2_, denoted by ×), a screening current I flowing around the loop, and a voltage V = f(Φ/Φ_0_) across the junction pair, periodically modulated by the externally applied magnetic field B, which creates magnetic flux lines (Φ) through the loop. The **right** panel shows the voltage–flux relationship (V vs. Φ/Φ_0_) for the SQUID, including the optimal working point (maximum dV/dΦ) and the voltage swing (ΔV). The relevant device parameters are shown in the Table below: Φ_0_ = h/2e ≈ 2.07 × 10^−15^ Wb; magnetic sensitivity ~1 fT/√Hz (fT: femtotesla); and operating temperature T_op_ < 4.2 K (cryogenic).

Figure 2 provides a cross-section of a SQUID for medical usage, offering a relatively complete representation of its architecture, which consists of a superconducting loop with two Josephson junctions located at specified points in the circuit. The diagram also shows magnetic flux lines directing their way through the loop and/or path to illustrate the quantum interference that provides the sensitivity to magnetic fields down to the femtotesla level.

When we describe sensor sensitivity in femtoteslas per square root hertz (fT/√Hz), we refer to the noise power spectral density and how the noise power is distributed across frequencies. This metric enables a fair comparison of sensors across diverse bandwidths and provides critical insights into the signal-to-noise ratios achievable in cardiac magnetic field measurements. The theoretical basis for quantum-enhanced cardiovascular diagnostics is underpinned by the fact that cardiac electrical activity generates magnetic fields that are not attenuated by body tissues, unlike when measuring electrical signals using traditional electrocardiography. This inherent physical advantage, coupled with the exceptional sensitivity of quantum sensors, enables non-invasive cardiac imaging with improved sensitivity and reduced tissue artifacts [9]. Most recent detailed reviews in Nature Reviews Physics have identified two principal quantum sensing modalities of particular relevance to cardiovascular science: optically pumped atomic magnetometers (OPMs) and nitrogen-vacancy (NV) centers in diamonds [10].

Optically pumped magnetometers are the most advanced quantum sensing technology currently being developed for clinical readiness in cardiovascular applications. These sensors detect the precession of atomic spins due to the presence of a magnetic field with sensitivities in the vicinity of femtoteslas per square root hertz. The clear advantage of OPMs for cardiovascular applications is that they operate without cryogenic cooling requirements, which have traditionally limited the capabilities of superconducting quantum interference devices (SQUIDs) used clinically [11]. As a consequence, OPM technology has enabled the advancement of wearable sensor arrays that measure cardiac signals while normal patients are moving and behaving normally—a far cry from the previous expected static or heavily shielded systems for magnetocardiography. A concise cross-modal comparison of quantum technologies, AI approaches, and conventional cardiovascular diagnostics is summarized in Table 2**.**

**Figure 3:**
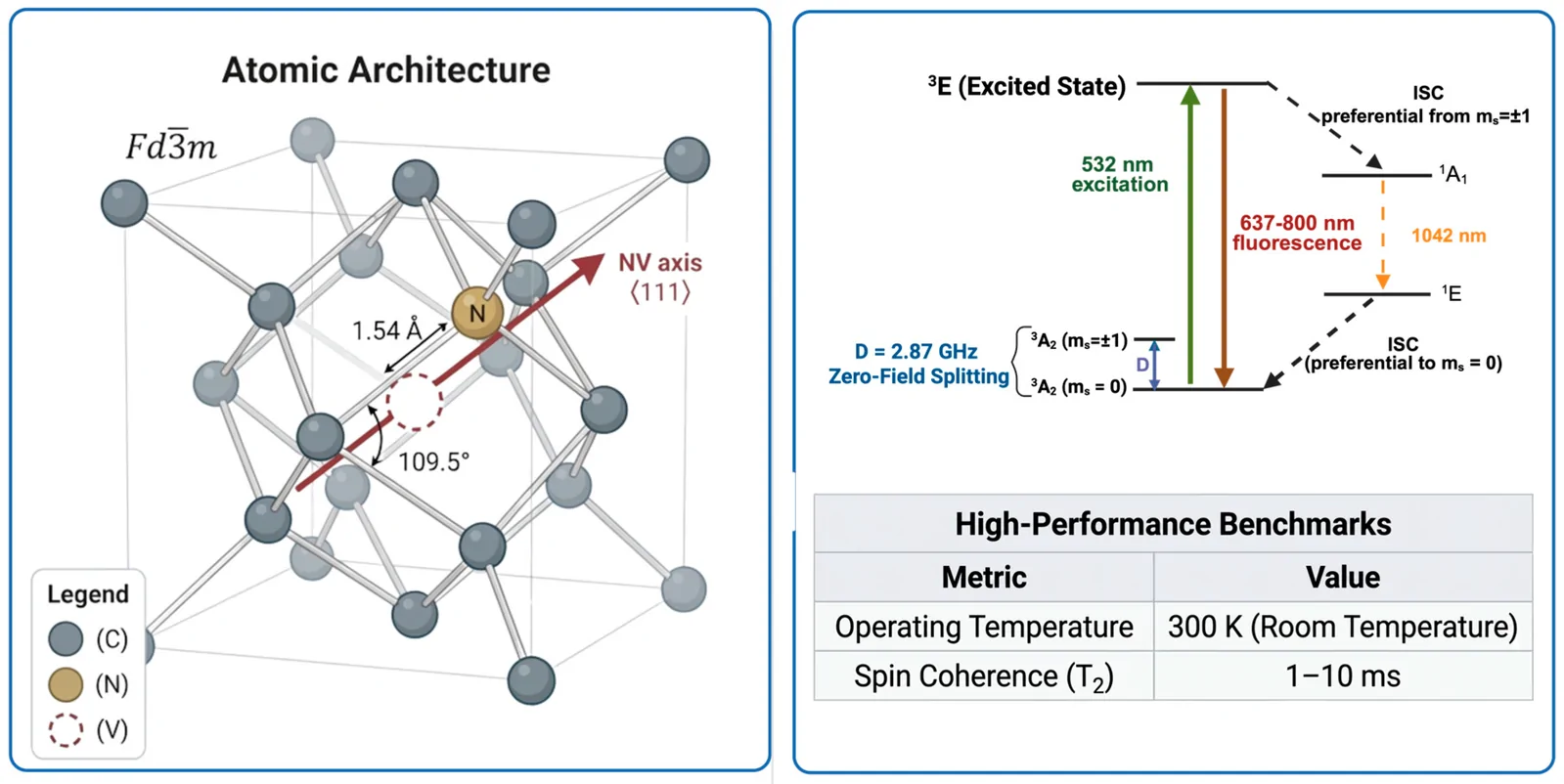
Atomic structure of nitrogen-vacancy center in diamond and energy level transitions for quantum sensing. **Left** panel: nitrogen-vacancy (NV^−^) defect in the diamond cubic lattice (Fd $\bar{3}$ m). A substitutional nitrogen atom (N, gold) is adjacent to a single carbon vacancy (V, dashed circle) along the ⟨111⟩ crystallographic direction. Carbon atoms (C, gray) are arranged in a tetrahedral lattice, forming bonds 1.54 Å in length and 109.5° in angle. **Right** panel: optical spin-polarization and readout cycle of the NV^−^ center at zero magnetic field: light at 532 nm excites the system from the Spin-Triplet Ground State ^3^A_2_ to the Excited State ^3^E; fluorescence emitted at wavelengths between 637 and 800 nm; zero-field splitting (D = 2.87 GHz) of the ground state creates two energy levels: m_s_ = 0 and the doubly degenerate m_s_ = ±1; population of the m_s_ = 0 substate is preferential due to Intersystem Crossing (ISC) pathways through the singlet states ^1^A_1_ and ^1^E, which are coupled by the 1042 nm infrared transition; enables optical readout of spin-state. Relevant Performance Metrics for NV^−^ are listed in the Table below: operating temperature = 300 K (room temperature); coherence time of the Spin State (T_2_): 1–10 ms.

Nitrogen-vacancy (NV) centers in diamond can operate at room temperature because of their unique quantum characteristics. As depicted in Figure 3, an NV center consists of a substitutional nitrogen atom adjacent to a lattice vacancy within the diamond crystal, the defect structure that underpins the room-temperature spin-triplet ground state used for magnetometry. NV centers have a spin-triplet ground state with a long coherence time (milliseconds), also at room temperature. This is due in part to the highly rigid diamond lattice, which helps protect the spin state. The zero-field splitting (2.87 GHz) that protects the spin states is large enough that thermal fluctuations at room temperature (kT ≈ 25 meV) are not enough to mix the spin states or destroy the quantum coherence necessary for sensing (see Figure 3).

Nitrogen-vacancy centers in diamond represent a new quantum sensing platform with unique advantages for cardiovascular applications that require high spatial resolution. Many quantum defects in diamond lattices can measure magnetic fields with nanoscale resolution at ambient temperatures and pressures. The range of potential applications of NV-center magnetometry in cardiovascular medicine extends beyond classic cardiac imaging, and even new subcellular-resolution studies of cardiac electrophysiology and the measurement of magnetic biomarkers of cardiovascular disease are also possible [5]. The atomic-scale properties of NV centers enable unprecedented spatial resolution, allowing for dimensions to be reduced to the point where it is even possible to create signatures from an individual cardiac cell and possibly subcellular volumes.

### 3.2. Clinical Applications and Real-World Implementations

The translation of quantum sensing technologies from laboratory demonstrations to clinical use has accelerated in recent years, with several systems now undergoing clinical validation studies. The most mature subset of applications involves magnetocardiography (MCG), where quantum sensors measure the magnetic fields associated with electrical activity in the heart. This differs from conventional electrocardiography (ECG), which measures electrical potentials that are reduced and distorted when traveling through body tissues. MCG measures the magnetic fields generated by the heart, which are minimally affected by the biological medium [12].

It is crucial to understand how quantum MCG compares to current cardiac imaging modalities. Cardiac magnetic resonance (CMR) is a powerful tool for evaluating anatomy and function, as well as providing tissue characterization. Speckle tracking echocardiography, on the other hand, allows for real-time assessment of myocardial strain. Quantum MCG contributes to the complement of tools used to assess the heart, providing unique electrophysiological information that is not available with structural imaging. This is particularly important in being able to detect electrical abnormalities that may occur at early stages, where no structural change has become evident.

In scenarios involving acute coronary syndromes, where timing is crucial, quantum MCG holds promise as an advantage over conventional diagnostic assessment methods. Magnetic field detection, as used in MCG, is a non-contact measurement. The ability to assess patients without placing electrodes offers the opportunity to reduce the time to diagnosis and the time and friction associated with electrode placement. Initial studies have demonstrated that quantum MCG can detect myocardial ischemia within a few minutes; however, larger clinical trials are necessary to evaluate the role of quantum MCG in emergency settings fully [13,14]. Cardiac implantable electronic devices (CIEDs), such as pacemakers and implantable cardioverter-defibrillators (ICDs), add a unique layer of complexity to quantum sensing. These devices create magnetic fields and can interfere with MCG measurements. Research continues to focus on developing and implementing algorithms that filter signals attributed to these devices but enhance relevant cardiac magnetic field information. Periodically, some quantum sensors may have to operate at a specific frequency or have adapted signal processing to maintain the relative fidelity of measurements taken from implanted devices [15].

Emerging clinical studies show superior diagnostic outcomes for quantum-enhanced MCG systems when compared to conventional cardiac diagnostic modalities. The MagMa Study, an extensive clinical study using quantum magnetocardiography (MCG) for non-ischemic cardiomyopathy, recorded an impressive sensitivity of 94.74% and specificity of 98.54% to cardiomyopathy [16]. These differences reflect improvements to conventional diagnostic approaches, for which quantum MCG systems would be especially valuable for cardiac abnormalities where minor clinical significance often means they go undetected with accepted forms of diagnosing cardiomyopathy pathologies.

Beyond improved diagnostic benefits, the clinical advantages of quantum MCG include improved patient comfort and accessibility. Unlike SQUID-based magnetocardiography systems, which require patients to remain still in a magnetically shielded space, next-generation quantum sensors can operate in environments outside of magnetic shielding and allow for patient movement during recording [11]. This capability is useful for pediatrics, especially for children who require it, and for patients with limited ability to sit still for the periods typically expected in conventional diagnostic procedures.

## 4. Quantum Machine Learning in Cardiovascular Risk Prediction and Diagnosis

### 4.1. Theoretical Foundations and Quantum Advantages

The rigorous evaluation of quantum sensing technologies in healthcare involves comparing them with existing diagnostic methods to prove their technical superiority and practical value in clinical settings. Various recent research reports have consistently shown that quantum-enhanced diagnostic tools surpass methods in terms of sensitivity, specificity, and accuracy [17]. Yet the extent of these enhancements varies based on the medical scenario under consideration and the group of patients under examination.

In the field of identifying heart rhythms (arrhythmia), advanced machine learning techniques influenced by quantum computing have shown some promising enhancements compared to traditional methods. A detailed research study published in Nature Scientific Reports revealed that machine learning algorithms enhanced with quantum technology achieved a 0.6% increase in accuracy over conventional methods and reduced training durations to 192 milliseconds [17]. Given the minor absolute differences and lack of prospective validation, these results should be treated as proof-of-concept without current clinical implications.

The effectiveness of magnetocardiography in diagnosing heart irregularities has shown remarkable results in situations where high sensitivity is crucial. The use of Cardiac Quantum Spectrum (CQS) technology, which analyzes quantum spectra from data, has proven to be more sensitive in detecting coronary artery disease compared to the standard 12-lead ECG methods. In trials, this tech managed to spot all patients with slow flow syndrome and found heart damage in patients with minor artery blockages, as per regular tests. This suggests that quantum-powered analysis can uncover important issues that are overlooked by traditional diagnostic methods.

Quantum sensing technologies are being tested in various settings to assess their effectiveness and identify potential limitations and issues for use in healthcare practices. One major concern is the interference that arises in hospitals due to electromagnetic noise from medical devices, affecting the performance of quantum sensors [18]. Researchers have created algorithms and AI-based methods to reduce this noise and improve the reliability of sensing systems in real-life medical environments; however, there is ongoing research to enhance their effectiveness further.

**Figure 4:**
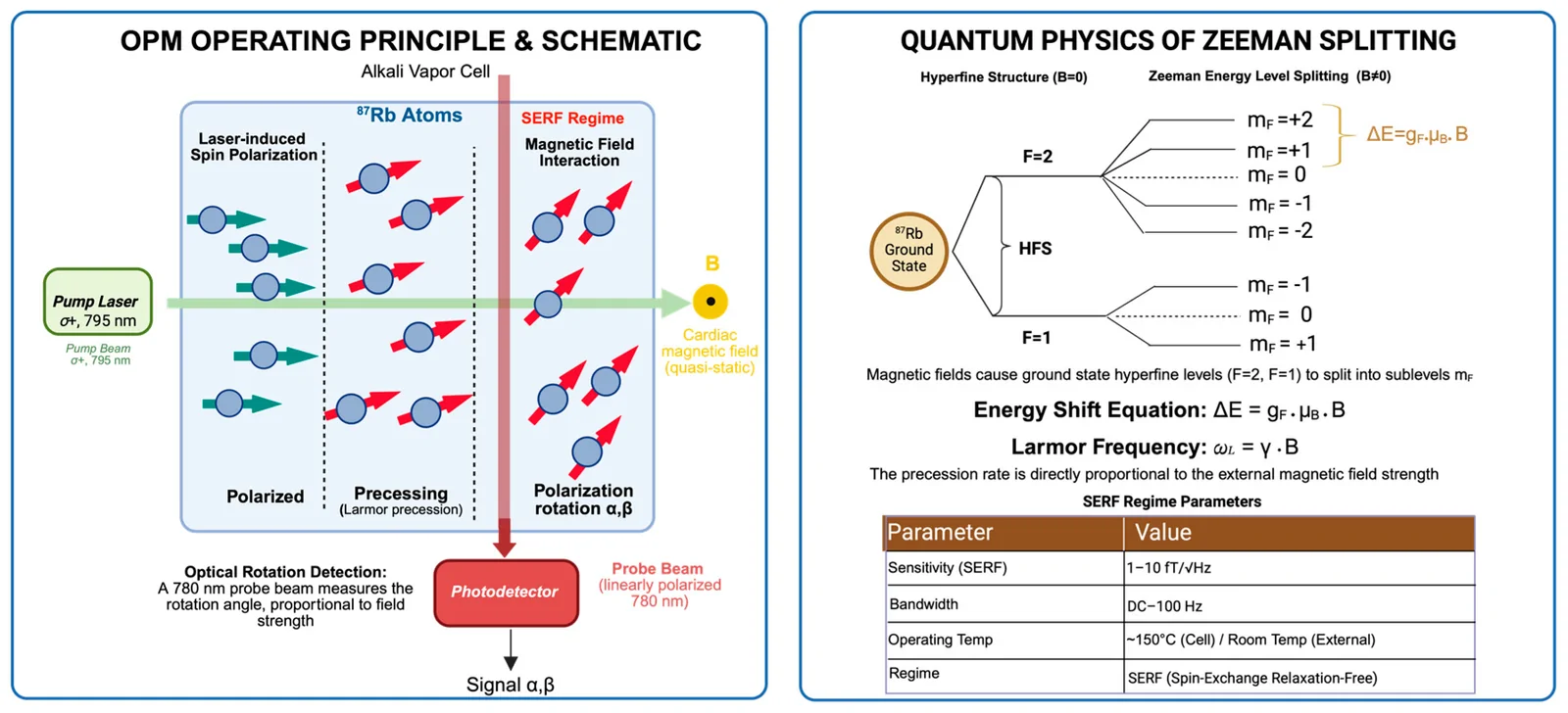
The Optically Pumped Magnetometer (OPM) operating principle and quantum physics of Zeeman splitting in the SERF regime. **Left** panel: a circularly polarized pump beam (σ^+^, 795 nm) generates spin polarization of ^87^Rb atoms contained within the alkali vapor cell; a linearly polarized probe beam (780 nm) crosses the cell perpendicular to the pump beam, through the polarization-rotation region, thereby generating an optical rotation signal (α, β) measured at the photodetector that is proportional to the quasi-static external cardiac magnetic field (B); the three labeled regions—polarized, precessing (Larmor precession), and polarization rotation—mark successive stages in the evolution of the spin ensemble along the pump direction. **Right** panel: an externally applied magnetic field (B) splits the hyperfine states of the ^87^Rb ground state due to the Zeeman effect, resulting in energy shifts in the hyperfine states (ΔE = g_F_·μ_B_·B) with the Larmor frequency ω_L_ = γ·B being directly proportional to the field strength. This represents the basic principle of the OPM measurement. Relevant Performance Metrics for the SERF regime are listed in the table below: sensitivity = 1–10 fT/√Hz (fT: femtotesla); bandwidth = DC–100 Hz; operating temperature ≈ 150 °C (cell)/room temperature (external); regime = SERF (Spin-Exchange Relaxation-Free). Solid colored arrows indicate the optical beams (green: σ+ pump, 795 nm; dark red: 780 nm probe); the external magnetic field B is shown as a yellow circled dot, showing that it is directed out of the plane of the figure, perpendicular to both beams. The small arrows within the vapor cell represent atomic spin orientation, aligned in the polarized region and tilted during Larmor precession. In the right panel, horizontal lines denote the hyperfine/Zeeman energy sublevels m_F_; the m_F_ = 0 sublevel is drawn as a dashed line because it is not shifted to first order in B, and the slanted lines are guides connecting each hyperfine manifold F to its shifted sublevels. The m_F_ ordering is inverted between the F = 2 and F = 1 manifolds because the hyperfine Landé factor gF has opposite signs for F = 2 and F = 1.

Figure 4 shows the atomic-level quantum sensing mechanism of optically pumped magnetometers (OPM) under development for cardiovascular applications. The schematic presents alkali atoms within a sensing chamber, with the interaction of laser beam pumping and external magnetic fields. Atomic-level energy diagrams and representations illustrate different spin states, which is a quantum mechanical principle known as spin precession.

### 4.2. Clinical Applications and Performance Evaluation

The application of quantum machine learning in cardiovascular medicine has evolved from theoretical demonstrations to studies investigating clinically relevant features. Now, several systems exist that deliver measurable improvements over conventional approaches. The most studied candidate applications focus on predicting cardiovascular risk. Quantum-enhanced algorithms extract person-level data from patients and identify patients who are at high risk for cardiac outcomes. Many candidate applications possess data features that can benefit from quantum machine learning because they have specific, complex, multi-dimensional data features that quantum algorithms can exploit to reveal patterns that are not visible to conventional approaches. It is worth considering the potential clinical implications of the reported improvements. While the 0.6% improvement in accuracy cited in the studies was statistically significant, it could hardly be considered clinically meaningful in the vast majority of cardiovascular applications [19,20]. In practice, to achieve the clinical relevance threshold when implementing new diagnostic technology, clinicians typically require 5–10% improvements in diagnostic accuracy, particularly with technology that is going to add greater complexity and cost [21,22]. The number needed to diagnose (NND) with such minor improvements is astronomical; clinicians would have to screen thousands of patients to identify an additional case they would otherwise miss using conventional methods. The importance of considering quantum–classical hybrid approaches should also stem from a comparison of these methods not only with traditional diagnostic techniques, but also with state-of-the-art classical machine learning algorithms [20]. Recent advances in deep learning, particularly convolutional neural networks and Transformer architectures, have produced remarkable performance in cardiovascular diagnostics. The quantum benefits could be relatively slight; we need to consider the maturity and accessibility of existing AI/ML areas. To date, no study has shown robust clinical utility for QML in heart-disease prediction; reported effect sizes remain clinically non-meaningful [23]. Current QML studies report accuracy gains on the order of ~0.6 percentage points (occasionally higher in narrowly defined tasks) and shorter training times (≈192 ms) [17]. While these differences can be statistically significant, they are too small to meet pragmatic thresholds for clinical meaningfulness in cardiology and—given present-day complexity and cost—do not support deployment. Moreover, shorter model training time does not translate directly into patient benefit; what matters are prospectively validated clinical endpoints, decision impact, and cost-effectiveness. In light of these limitations, QML should presently be regarded as a research avenue rather than a ready-to-use clinical solution (see ‘Future Lines of Research’).

The prospect of quantum machine learning has also been demonstrated in a narrow sense in the value of identified features in civilization-specific cardiovascular applications, particularly arrhythmia classification and cardiac imaging analysis. A systematic review of quantum machine learning for cardiac arrhythmia classification demonstrated an improvement in accuracy associated with existing classical approaches. Most notably, quantum-enhanced analysis favored the detection of rare or other complex forms of arrhythmia that were not adequately managed by conventional algorithms. The quantum-enhanced models showed favorable parameters for classifying arrhythmias, distinguishing different arrhythmias, and identifying properties of patients at risk of sudden cardiac death [24].

### 4.3. Limitations and Challenges in Clinical Implementation

Despite the promising results demonstrated in research studies, the clinical implementation of quantum machine learning in cardiovascular medicine faces significant challenges that must be addressed for widespread adoption. The most fundamental limitation is the current state of quantum hardware, which remains noisy, error-prone, and limited in the number of qubits available for computation [25]. These hardware limitations constrain the complexity of problems that can be addressed using current quantum systems, necessitating the development of error-correction techniques and noise-resilient algorithms.

Therefore, they cannot be meaningfully classified or compared with cardiovascular applications. Most importantly, no peer-reviewed publication has reported improvements exceeding 1% in cardiovascular applications with any actual quantum hardware.

Scalability represents another significant challenge for quantum machine learning in cardiovascular medicine. Current quantum systems are limited in their ability to process the large datasets that are characteristic of modern cardiovascular research and clinical practice. Electronic health records, imaging databases, and genomic datasets often contain millions of patient records with thousands of features per patient, exceeding the current capabilities of quantum hardware [24]. The development of quantum algorithms that can efficiently process such large-scale datasets remains an active area of research.

The integration of quantum machine learning systems into existing clinical workflows presents additional challenges related to data security, regulatory approval, and clinical validation. Quantum computing systems require specialized infrastructure and expertise that may not be readily available in most clinical settings. The development of cloud-based quantum computing services may address some of these challenges, but concerns about data security and patient privacy must be carefully considered when using external quantum computing resources for clinical applications [26].

## 5. Quantum-Enhanced Drug Discovery and Therapeutic Applications

### 5.1. Quantum Computing in Cardiovascular Drug Development

Using quantum computing for drug discovery is the most promising application of quantum technologies in cardiovascular care in the long term. The fundamental aspect of drug discovery that poses a challenge is the inherently exponential complexity of molecular interactions. Every time a new molecular component or system is introduced, the number of potential molecular configurations and drug–target interactions increases exponentially [27]. Classically, we are limited in our attempts to synthesize these complex molecular systems and interactions, particularly for large proteins and various multi-target-based drug interactions that make up the patient population receiving cardiovascular therapeutics over their lifetime.

Although the recent discovery of quantum-enhanced drug discovery through KRAS inhibitors for cancer therapeutics demonstrates the potential for a method that could be applied to cardiovascular drug targets, this finding highlights the importance of further research in this area [28]. The same hybrid quantum–classical approach could be adapted for the design of inhibitors targeting cardiovascular diseases involving PCSK9 (cholesterol), factor Xa (anticoagulation), or angiotensin-converting enzyme (hypertension). However, to date, there have been no cardiovascular-specific quantum-enhanced kinetic drug discovery successes published.

This landmark study takes a quantum–classical hybrid generative model approach that includes quantum circuit Born machines (QCBMs) being used with traditional long short-term memory (LSTM) networks to generate new molecular structures. The quantum portion is being run in a 16-qubit quantum processor, which is utilized to generate complex distributions of probability and make it possible to explore the overall chemical space beyond the capabilities of traditional algorithms. They synthesized 15 of their generated compounds, including over 1 million of the generated compounds, and they reported 2 compounds of interest for further development as KRAS inhibitors [28].

The quantum advantage in drug discovery is both the ability of quantum systems to represent and manipulate the quantum mechanical properties that govern molecular interactions via their capabilities, inherent aspects of quantum systems, and their representation of phenomena in their native quantum representations. Quantum computers should logically be able to simulate a variety of molecular systems at the quantum level from within their native (quantum) worlds, changing how detailed drug–target interactions could be revealed as triggered or not be solvable entirely from within classical systems [29]. This is very significant for cardiovascular drug discovery, as understanding how these complex protein–drug interactions occur is critical to the development of more effective therapeutics with fewer side effects.

There has been considerable development of quantum algorithms for simulating molecular systems over the past few years, with various approaches demonstrating significant promise in their applications to cardiovascular molecular interactions. Variational quantum eigensolver (VQE) techniques have been developed to calculate molecular ground states and excited states, allowing for the prediction of drug binding affinity and drug reaction pathways of interest [30]. In theory, these quantum algorithms could provide expedient, specific, and accurate forecasts for drug efficacy and toxicity compared to evaluations using classical approaches, providing more thorough means of drug development and less difficulty overall with attrition of new agents through development or trial failures.

### 5.2. Quantum Molecular Modeling and Protein–Drug Interactions

As already mentioned, the application of quantum computing to molecular modeling for cardiovascular medicine specifically relates to understanding the many complex features associated with the binding of therapeutic drugs to cardiac proteins. Chemical bonding and interactions are naturally quantum chemical processes. Quantum computers are uniquely capable of computing the interactions and generating insights related to molecular binding with greater accuracy than classical approximations can provide [31]. The quantum advantage can be large for cardiovascular drug discovery because very small differences in the ways molecular interactions occur can mean the difference between therapeutic success and unwanted negative side effects.

Recent developments in quantum molecular simulation have demonstrated the ability to model protein–drug interactions with unparalleled accuracy. Quantum algorithms can incorporate quantum effects, including tunneling, superposition, and entanglement, which play important roles in molecular recognition and binding [32]. Drug selectivity and specificity are fundamental to attaining an efficacious therapeutic effect while avoiding undesirable off-target effects that could ultimately impair cardiac function, and they are sensitive to quantum effects.

The development of quantum-enhanced molecular dynamics simulations broadens possibilities, providing novel ways to elucidate the time-dependent evolution of protein–drug complexes, including the speed and rate of drug binding and release, whether binding is accompanied by conformational changes in the original protein, and any dynamic stability of the drug–protein complex over time [33]. The understanding of how a drug interacts with a target protein at the molecular level over time can be used to inform optimal drug design and subsequent therapy.

Quantum machine learning approaches were also developed and used to predict drug–target interactions and elucidate molecular properties for cardiovascular therapeutics. Such approaches provide a fusion of quantum computing with machine learning algorithms to capitalize on patterns within a database of molecular data that signify therapeutic activity [34]. Quantum-enhanced pattern recognition capabilities could provide insights into drug targets and offer early indications of the cardiovascular implications of new therapeutic drugs.

## 6. Current Challenges and Limitations

### 6.1. Technical and Hardware Limitations

The clinical use of quantum technologies for cardiovascular medicine will present remarkable technically induced challenges before practicality may become commonplace. Currently, quantum hardware remains in the noisy intermediate-scale quantum (NISQ) era, in which systems have a limited number of qubits and are prone to noise, restricting their practical applications [35]. This has significant implications for how complex cardiovascular problems can be solved with current quantum systems. For quantum machine learning-based applications in predictive heart disease, any modest improvement in accuracy must be balanced with the high technical complexity and infrastructure requirements of quantum systems [23]. The economic barriers to the adoption of quantum technologies for cardiovascular medicine are almost insurmountable. Traditional SQUID-based MCG systems can cost anywhere from USD 1–3 million, requiring specific shielded rooms (estimated at USD 500,000–1 million added cost).

In contrast, a high-end 12-lead ECG may cost USD 20,000–50,000, while advanced cardiac MRI systems range from approx. USD 1 to 3 million and primarily serve to obtain diagnostic meaning and clinical sense.

The clinical usefulness of quantum-enhanced diagnostic testing must be carefully evaluated within traditional epidemiological frameworks of real-world impact. The Number Needed to Screen (NNS) from Rembold provides an analytical approach to determine how many patients need to be screened or tested to find one additional true positive compared to various diagnostic options (e.g., Aspirin) [36]. NNS also helps assess whether a minor difference in diagnostic performance or actionability justifies large capital investments in new technologies.

The NNS for quantum-enhanced cardiovascular diagnostics is computed using Rembold’s formula, as follows:

NNS = 1/[(Sensitivity_2_ − Sensitivity_1_) × Prevalence]

where Sensitivity_2_ is the performance of the quantum-enhanced test, Sensitivity_1_ is the performance of the standard test, and Prevalence indicates the proportion of the disease in the tested population [36]. This calculation assumes equal specificity for both tests. If specificity differs, the analysis becomes more complex, as accuracy can shift with changes in prevalence, patient population spectrum effects, and clinical characteristics [37].

Using data from the MagMa Study, the quantum magnetocardiogram (MCG) demonstrated 94.74% sensitivity and 98.54% specificity for detecting cardiomyopathy [16]. With these figures and typical sensitivity rates for conventional cardiac magnetic resonance (CMR) imaging of 80–85%, and assuming a 5% prevalence among high-risk screened groups—such as symptomatic patients, those with abnormal ECGs, or a family history—the NNS is calculated as 1/[(0.9474 − 0.80) × 0.05], which equals 136. This means 136 patients from the at-risk group would need to undergo quantum MCG screening to identify one case of cardiomyopathy that CMR might miss. The high specificity also reduces false positives (non-cardiomyopathy cases), thereby decreasing downstream costs. The clinical performance of quantum MCG reveals a significant improvement in diagnostic ability compared to conventional imaging methods. The 14.74 percentage point increase in sensitivity over CMR is a meaningful step forward in cardiomyopathy detection, especially for high-risk populations where early and accurate diagnosis can greatly influence patient outcomes. Practically, this enhanced sensitivity means about 15 more cases per 100 patients with cardiomyopathy could be identified, who might otherwise obtain false-negative results from standard CMR imaging. The number needed to screen calculation offers valuable insight into the clinical efficiency of using quantum MCG in targeted screening efforts. In high-risk groups, where disease prevalence reaches 5%, this technology shows its potential to find cases that might otherwise go unnoticed. The resulting NNS of 136 indicates a manageable screening effort, especially considering the serious risks of undiagnosed cardiomyopathy, such as sudden cardiac death, worsening heart failure, and blood clots. Additionally, the outstanding specificity of 98.54% makes quantum MCG a particularly useful diagnostic tool. This high specificity ensures minimal false positives, protecting patients from unnecessary psychological stress and avoiding extra costs for confirmatory tests and follow-up. The combination of higher sensitivity and high specificity makes quantum MCG a valuable addition to the tools available for cardiomyopathy screening in carefully chosen groups.

The economic considerations, which extend beyond initial test costs, are considerably more complex than NNS outcomes. Detecting cardiomyopathy earlier can delay disease progression, prevent heart failure and morbidity, reduce hospitalization, and improve long-term survival through better diagnosis and treatment—potentially avoiding substantial long-term costs and rehabilitation expenses. The additional cost per case identified is calculated as NNS × (cost of quantum test − cost of conventional test). For example, quantum MCG costs roughly USD 800 per test (assuming a reasonable amortization of a USD 100,000 optically pumped magnetometer), and CMR typically costs far more than USD 500. In most developed countries, real-world prices are usually USD 1000–5000 in the U.S. (Medicare ≈ USD 500–600 at the low end) and EUR 500–1300 in Europe, with private clinics or hospitals often charging several thousand (e.g., GBP 750–2500 in the UK) then the excess cost for 136 tests is 136 × (USD 800–500) = 40,800. This expense should be weighed against the probable USD 100,000 in costs associated with managing advanced cardiomyopathy over the patient’s remaining lifetime.

Currently, quantum magnetocardiography systems are capital-intensive, with optically pumped magnetometers starting at around USD 100,000 and whole SQUID (superconducting quantum interference device)-based systems nearing USD 1 million [38,39]. While technological advancements and economies of scale will help reduce these costs, conventional economic assessments should consider options like renting, leasing, or financing the upfront investments to make implementation more feasible [40,41]. The scalability problem with cardiovascular applications is particularly severe because clinical datasets often contain millions of patients, with thousands of parameters (or features) per patient record. Current quantum systems have limited datasets or resolutions that they can process, thus limiting their direct relevance to real-world clinical situations [42]. The exploration of quantum algorithms capable of managing large-scale cardiovascular datasets is ongoing, and the emergence of hybrid quantum–classical approaches shows the most promise for near-term applications, which should ultimately include both clinical reference/classical observational healthcare data and alternative clinical reference/quantum diagnostic data. Environmental noise and decoherence are intrinsic obstacles to quantum sensing applications in cardiovascular medicine. Hospital settings represent one of the most challenging environments for deploying quantum devices due to electromagnetic interference from medical devices, magnetic field fluctuations, and vibrations associated with physical structures [11]. Although advanced waveform signal-processing algorithms and AI-driven noise reduction techniques now exist to address environmental problems, the overall robustness of quantum sensing systems in actual clinical environments still needs to be realized. Fundamental physics will impose strict limitations while allowing for reasons to make informed assessments about exploring quantum cardiovascular applications. Decoherence rates increase with system size and temperature, making it exponentially more challenging to protect quantum states against decoherence. The no-cloning theorem prohibits the ability to make perfect copies of quantum states, which limits diagnostic or other applications and the feasibility of clinical use. The Heisenberg uncertainty principle establishes a fundamental limit where simultaneous measurements will inherently lack precision. Overall, these statements represent true laws of physics that do not exist as engineering issues to overcome, which will always limit or impact quantum-based medical applications.

### 6.2. Clinical Validation and Regulatory Challenges

The pathway to applying quantum technologies in clinical practice from research labs will include multiple phases of validation studies, as well as regulatory approval processes that are currently in development. The regulatory landscape for quantum medical devices is rapidly evolving. Regulatory agencies, including the FDA, are in the nascent stages of developing a framework for evaluating and approving quantum-enhanced diagnostics and therapeutic systems. The unique properties of quantum technologies compared to their classical counterparts (for example, the probabilistic nature of quantum technologies and their potential sensitivity to environmental conditions) will create new challenges in documentation and validation that may not be addressed by existing models for validating medical devices.

A cost-effectiveness analysis can be conducted using quality-adjusted life years (QALY) metrics, which are essential for evaluating healthcare technologies. Preliminary figures indicate that a quantum MCG would need to prevent at least 0.5 cardiovascular events per 100 patients screened to bring the cost per QALY below USD 100,000, the generally accepted threshold in developed healthcare systems for technologies to be considered for implementation. However, current evidence does not support this level of clinical benefit [43].

Clinical validation studies for quantum cardio technologies will need to show that the new technologies confer not only a technical and diagnostic advantage but also a clinical advantage and support cost-effectiveness analysis. The small advances in diagnostic accuracy from quantum systems must be translated into measurable outcomes for patients before clinical application can be justified [44]. Long-term follow-up studies are necessary to determine the impact of cardiovascular diagnostics enhanced with quantum technology on patient management and clinical outcomes.

The adoption of quantum technologies in clinical practice will be hindered by the need for healthcare providers to undergo training to operate these technologies. Then, issues with training, maintenance, and quality assurance would need to be addressed, which would be very demanding, particularly for biophotonics. The sophistication of quantum technologies may preclude their use in lower-resourced healthcare environments where access to post-graduate educational training may not exist [45]. Simple user interfaces and automated operator procedures are essential for the successful implementation of quantum cardiovascular technologies in clinical practice.

### 6.3. Economic and Accessibility Considerations

The high cost of quantum technologies presents a significant barrier to their application in cardiovascular medicine. Currently available quantum systems require dedicated infrastructure, such as cryogenic cooling systems, electromagnetic shielding, and advanced electronic control, which contribute to high capital and operational expenditure costs [46]. Any cost–benefit analysis of quantum cardiovascular technologies needs to include ongoing maintenance, training, and upgrade costs in addition to the original capital cost.

In the early stages of application, access to quantum cardiovascular technologies will likely be limited to large medical centers and research institutions with the infrastructure and skill to set up, maintain, and implement these systems. Limited access could deepen existing inequalities in healthcare and further marginalize the most vulnerable populations. These populations traditionally have limited access to care and optimum health outcomes without advances from quantum-enhanced diagnostic and therapeutic evaluation [47]. Strategies to bridge this gap are being evaluated, including the use of quantum technologies as a cloud service and the development of portable quantum sensors that are accessible to marginalized communities. The economic burden of quantum technologies in cardiovascular medicine raises distinct ethical questions regarding equity in the healthcare system before the complete adoption of quantum diagnostic tools is pursued for patient use. If quantum diagnostics is better than the standard of care but is only available to wealthy patients or patients in developed countries, quantum diagnostics can worsen health inequities and redistribute healthcare benefits toward the already privileged. This raises a significant question for healthcare systems: Is it ethical to pursue slight advances in diagnosis and treatment of individual patients when basic cardiovascular care access is not routinely accessible to many others? The economic implications of quantum cardiovascular medicine encompass the costs society incurs that are directly related to healthcare, also allowing for a critical analysis of possible positive impacts on society, including increased productivity, reduced disability, and an improved quality of life. There is a growing need for economic modeling studies to analyze broader considerations and inform healthcare policy regarding the adoption and reimbursement of quantum cardiovascular technologies.

## 7. Future Directions and Emerging Opportunities

### 7.1. Next-Generation Quantum Sensing Platforms

The future of quantum sensing in cardiovascular medicine will focus on next-generation platforms that address current limitations and enhance capabilities. New quantum sensing innovations, such as room-temperature quantum sensors based on defects in silicon carbide and atomic vapor cells, could bypass the need for cryogenic temperatures that have limited the acceptance of traditional quantum sensors in the clinic [48]. These types of new platforms will lead towards the vision of portable, low-cost quantum cardiovascular diagnostics for point-of-care applications. The combination of quantum sensor devices with AI and machine learning is an exciting and compelling path for cardiovascular medicine [10]. AI-enabled quantum sensors might allow for real-time analysis of cardiac signals, automatic identification of abnormalities, and predictive modeling for cardiovascular events. By combining the high precision of quantum sensing devices with the AI data-rich classifying power from the sensors, the ultra-sensitivity of quantum sensing will ultimately provide the detection of subtle cardiovascular deviations that are only possible through conventional clinical means. The innovations in quantum sensor arrays and networking can potentially yield full cardiovascular behavior monitoring systems that would provide rich spatiotemporal data on cardiac function. Multi-sensor quantum arrays can monitor electrical, magnetic, and mechanical changes in cardiac activity concurrently and provide holistic representations of cardiovascular health.

### 7.2. Quantum-Enhanced Personalized Medicine

Quantum computing-based personalized cardiovascular medicine holds true potential for radically transforming patient care. Quantum algorithms could examine large multi-omics datasets, which include genomic, proteomic, and metabolomic data, in order to derive personalized risk profiles and treatment recommendations. The scaling features of quantum computing may enable an entire dimension of data analysis necessary for true personalized medicine [49].

Quantum machine learning algorithms may further identify subtle deviations in patient data that indicate an increased risk for cardiovascular disease or a response to treatment. The power of quantum-based machine learning algorithms may identify new biomarkers and therapeutic targets that are missed with classic methods [50]. Quantum-enhanced clinical decision support programs might mean that clinicians would be able to provide more thoughtful and personalized recommendations regarding cardiovascular care.

Combining quantum sensing with personalized medicine (patient preferences, patient genetics, and multi-omics data) may provide the ability to continuously and non-invasively monitor each patient’s response to cardiovascular interventions in real time. Quantum sensors could provide continuous, non-invasive monitoring of cardiac parameters so that providers could alter the treatment protocol based on each patient’s unique responses. Such an approach to cardiovascular care could improve efficacy and safety while minimizing adverse effects and improving outcomes for individual patients.

### 7.3. Quantum Biology and Fundamental Understanding

The new area of quantum biology can provide new insights into the basic mechanisms of cardiovascular function and dysfunction. Recent theoretical work demonstrates that cardiac ion channels and the electrophysiology of the heart have quantum effects, which could lead to completely new ways to study and treat cardiovascular disease. There is discussion about the functional relevance of quantum effects in biological systems. Still, research in this area may allow us to identify new targets in disease and new modes of intervention.

## 8. Emerging Ideas/Controversies

Although much of this review has concentrated on quantum cardiovascular technologies that are already in use or close to clinical adoption, there are also strands of research that are far more tentative. These speculative ideas are worth outlining because they help shape the discussion about where the field might go next, even if they are not ready for the clinic.

### 8.1. Quantum Biology

Several modeling studies have suggested that quantum tunnelling of sodium, potassium, or proton ions through cardiac ion channels could, at least in theory, affect excitability and possibly trigger arrhythmias [50,51]. These effects are thought to be more likely under certain pathological conditions such as acidosis or hyperexcitability. However, no experiment has yet shown them to occur in the human heart. Classic work on ion channel gating [52,53] reminds us how complex these proteins are in excitable membranes, including those in cardiomyocytes.

Quantum effects have been observed in other living systems. The best-known examples are coherent energy transfer in photosynthesis [54] and magnetoreception in birds [55]. These examples are not cardiac, and the conditions in which they occur are very different, but they do show that biology can sometimes sustain quantum behavior.

Some researchers have proposed combining these quantum biology ideas with modern quantum sensing. In theory, this could make it possible to detect extremely subtle “quantum signatures” in cardiac electrophysiology that would be invisible to classical instruments [10]. It is an intriguing possibility, but without convincing experimental proof, it must remain a long-term goal.

### 8.2. Quantum Machine Learning at the Bedside

Hybrid quantum–classical algorithms have shown small, but measurable, accuracy gains over strong classical methods in retrospective cardiovascular datasets—about 0.6 percentage points in most reports [17,23]. Training times are shorter as well. The real question, though, is whether these marginal differences will translate into better patient care. So far, there is no such evidence. It will take prospective trials to know whether QML can genuinely improve decision-making or outcomes [24]

### 8.3. Future Sensing Platforms and Personalization

Some of the most ambitious proposals involve ideas such as population-scale quantum sensor networks for continuous heart monitoring [10], using multi-omics data in quantum-enhanced personalized medicine [49], and developing quantum simulations specifically for cardiovascular biology [27,29]. These could make earlier diagnosis possible and allow treatment to be adapted more precisely to each patient. They might also offer new insights at the population level. But for now, they remain in preliminary stages, facing major challenges in scale-up, regulatory approval, data security, and cost-effectiveness [26,46].

## 9. Conclusions

Quantum sensing—most notably magnetocardiography—shows the most credible near-term value, pending larger trials. Quantum-enhanced machine learning yields marginal, as-yet clinically unproven gains, and quantum drug discovery in cardiology remains preclinical. The next step is rigorous, multicenter validation with standardization, head-to-head comparators, and health-economic/regulatory alignment. Until then, this technology should be adopted selectively and should remain patient-centered in judging real-world value.

## Figures and Tables

**Table 1:** Inclusion and exclusion criteria with frequent reasons for exclusion.

Inclusion Criteria	Exclusion Criteria	Frequent Reasons for Exclusion
Application of quantum physics concepts to cardiovascular medicine Use of quantum-based technology for diagnosis or therapy Theoretical models describing quantum mechanisms in cardiology Systematic reviews on quantum applications in cardiovascular medicine Conference papers with adequate methodological detail	Studies with no quantum element or no cardiovascular relevance Full text or English abstract unavailable Opinion pieces/editorials with no original data Duplicate publications	No cardiovascular focus Mislabeled as ‘quantum’ but using only classical methods No quantitative data or methodological description Low methodological quality or retracted Duplicate dataset already included

**Table 2:** Comparison of quantum technologies, artificial intelligence, and conventional methods in cardiovascular diagnostics.

Domain	Quantum Technology Example	Classical/AI Equivalent	Quantum Advantage	Current Limitations/ Stage
Sensing	SQUID-based MCG ^1^; OPM ^2^; NV centers ^3^	ECG, MRI, CT	Ultra-high sensitivity (fT range) ^4^; non-contact measurement, direct cardiac magnetic field detection	High cost (USD 100 k–1 M), specialized infrastructure, limited clinical validation studies
Machine Learning	Quantum–classical hybrid models for risk prediction	Classical ML/DL (Random Forests, CNNs, Transformers)	Marginal accuracy gains (typically ≤ 1%; occasionally higher in lab settings); faster training	Minimal clinical benefit; training speed does not translate to patient outcomes; NISQ and hardware constraints
Molecular Simulation	Variational Quantum Eigensolver (VQE), QCBM	Classical molecular dynamics, density functional theory	Potential for accurate simulation of complex molecular interactions; theoretical advantages for drug-target modeling	Experimental phase; no validated clinical applications; exceeds current quantum hardware capabilities

Table Notes: ^1^ SQUID: Superconducting Quantum Interference Device. ^2^ QPM: Optically Pumped Magnetometer. ^3^ NV: Nitrogen-vacancy centers in diamond. ^4^ T: femtotesla (10^−15^ Tesla); Earth’s magnetic field ≈ 50 μT (50 × 10^−6^ Tesla).

## Abbreviations

The following abbreviations are used in this manuscript:

AI	Artificial Intelligence
CardiAQ	Cardiac Quantum (diagnostic platform)
CC BY	Creative Commons Attribution
CIED	Cardiac Implantable Electronic Device
CMR	Cardiac Magnetic Resonance
CNN	Convolutional Neural Network
CQS	Cardiac Quantum Spectrum
CT	Computed Tomography
CVD	Cardiovascular Disease
DL	Deep Learning
ECG	Electrocardiogram
fT/√Hz	Femtoteslas per square root hertz
GBD	Global Burden of Disease
GHz	Gigahertz
ICD	Implantable Cardioverter-Defibrillator
kT	Boltzmann constant times temperature
KRAS	Kirsten rat sarcoma viral oncogene homolog
LSTM	Long Short-Term Memory
MCG	Magnetocardiography
meV	Millielectron volt
MRI	Magnetic Resonance Imaging
ML	Machine Learning
NND	Number Needed to Diagnose
NNS	Number Needed to Screen
NISQ	Noisy Intermediate-Scale Quantum
NV	Nitrogen-Vacancy
OPM	Optically Pumped Magnetometer
PCSK9	Proprotein convertase subtilisin/kexin type 9
QALY	Quality-Adjusted Life Year
QCBM	Quantum Circuit Born Machine
QML	Quantum Machine Learning
QuEL	Quantum-Enhanced Learning
SQUID	Superconducting Quantum Interference Device
VQE	Variational Quantum Eigensolver

## References

[1] World Health Organization (2025). Cardiovascular Diseases (CVDs).

[2] Roth G.A., Mensah G.A., Johnson C.O., Addolorato G., Ammirati E., Baddour L.M., Barengo N.C., Beaton A.Z., Benjamin E.J., Benziger C.P. (2020). Global Burden of Cardiovascular Diseases and Risk Factors, 1990–2019: Update from the GBD 2019 Study. J. Am. Coll. Cardiol..

[3] Biamonte J., Wittek P., Pancotti N., Rebentrost P., Wiebe N., Lloyd S. (2017). Quantum machine learning. Nature.

[4] Wang J., He F., Sun S. (2023). Construction of a new smooth support vector machine model and its application in heart disease diagnosis. PLoS ONE.

[5] Barry J.F., Schloss J.M., Bauch E., Turner M.J., Hart C.A., Pham L.M., Walsworth R.L. (2020). Sensitivity Optimization for NV-Diamond Magnetometry. Rev. Mod. Phys..

[6] Page M.J., McKenzie J.E., Bossuyt P.M., Boutron I., Hoffmann T.C., Mulrow C.D., Shamseer L., Tetzlaff J.M., Akl E.A., Brennan S.E. (2021). The PRISMA 2020 statement: An updated guideline for reporting systematic reviews. BMJ.

[7] Newcastle-Ottawa Quality Assessment Form for Cohort Studies. n.d. https://www.google.com/url?sa=i&url=https%3A%2F%2Fwww.ncbi.nlm.nih.gov%2Fbooks%2FNBK115843%2Fbin%2Fappe-fm3.pdf&psig=AOvVaw13n6U7mXyU6kiP_y61cQc8&ust=1755783180184000&source=images&cd=vfe&opi=89978449&ved=0CAYQrpoMahcKEwiwtq6XwJmPAxUAAAAAHQAAAAAQBA.

[8] Ling S.J., Moebs W., Sanny J. (2016). Superconductors. University Physics.

[9] Budker D., Romalis M. (2006). Optical Magnetometry. Nat. Phys..

[10] Aslam N., Zhou H., Urbach E.K. (2023). Quantum sensors for biomedical applications. Nat. Rev. Phys..

[11] Boto E., Holmes N., Leggett J., Roberts G., Shah V., Meyer S.S., Muñoz L.D., Mullinger K.J., Tierney T.M., Bestmann S. (2018). Moving magnetoencephalography towards real-world applications with a wearable system. Nature.

[12] Sternickel K., Braginski A.I. (2006). Biomagnetism using SQUIDs: Status and perspectives. Supercond. Sci. Technol..

[13] Her A.Y., Dischl D., Kim Y.H., Kim S.W., Shin E.S. (2023). Magnetocardiography for the detection of myocardial ischemia. Front. Cardiovasc. Med..

[14] Pena M.E., Pearson C.L., Goulet M.P., Kazan V.M., DeRita A.L., Szpunar S.M., Dunne R.B. (2020). A 90-second magnetocardiogram using a novel analysis system to assess for coronary artery stenosis in Emergency department observation unit chest pain patients. IJC Heart Vasc..

[15] Özkartal T., Demarchi A., Caputo M.L., Baldi E., Conte G., Auricchio A. (2022). Perioperative Management of Patients with Cardiac Implantable Electronic Devices and Utility of Magnet Application. J. Clin. Med..

[16] Suwalski P., Wilke F., Latinova E., Paci G., Satilmis G., Klingel K., Kelle S., Weiner J., Beule D., Lüscher T.F. (2025). The MagMa Study: Quantum Magnetocardiography in Non-Ischemic Cardiomyopathy. medRxiv.

[17] Babu S.V., Ramya P., Gracewell J. (2024). Revolutionizing heart disease prediction with quantum-enhanced machine learning. Sci. Rep..

[18] Smith F.E., Langley P., van Leeuwen P., Hailer B., Trahms L., Steinhoff U., Brouke J.P., Murray A. (2006). Comparison of magnetocardiography and electrocardiography: A study of automatic measurement of dispersion of ventricular repolarization. Europace.

[19] (2025). New Medical Services and New Technologies.

[20] Larner A.J. (2018). Number Needed to Diagnose, Predict, or Misdiagnose: Useful Metrics for Non-Canonical Signs of Cognitive Status?. Dement. Geriatr. Cogn. Dis. Extra.

[21] Butler J., Khan M.S., Mori C., Filippatos G.S., Ponikowski P., Comin-Colet J., Roubert B., Spertus J.A., Anker S.D. (2020). Minimal clinically important difference in quality of life scores for patients with heart failure and reduced ejection fraction. Eur. J. Heart Fail..

[22] Spertus J.A., Jones P.G., Sandhu A.T., Arnold S.V. (2020). Interpreting the Kansas City Cardiomyopathy Questionnaire in Clinical Trials and Clinical Care: JACC State-of-the-Art Review. J. Am. Coll. Cardiol..

[23] Kumar A., Dhanka S., Sharma A., Bansal R., Fahlevi M., Rabby F., Aljuaid M. (2025). A hybrid framework for heart disease prediction using classical and quantum-inspired machine learning techniques. Sci. Rep..

[24] Gupta R.S., Wood C.E., Engstrom T., Pole J.D., Shrapnel S. (2025). Quantum Machine Learning for Digital Health? A Systematic Review. npj Digit. Med..

[25] Aaronson S. (2008). The Limits of Quantum Computers. Sci. Am. Mag..

[26] Chow J.C.L. (2024). Quantum Computing in Medicine. Med. Sci..

[27] Cao Y., Romero J., Olson J.P., Degroote M., Johnson P.D., Kieferová M., Kivlichan I.D., Menke T., Peropadre B., Sawaya N.P. (2019). Quantum Chemistry in the Age of Quantum Computing. Chem. Rev..

[28] Ghazi Vakili M., Gorgulla C., Snider J., Nigam A., Bezrukov D., Varoli D., Aliper A., Polykovsky D., Padmanabha Das K.M., Cox H. (2025). Quantum-computing-enhanced algorithm unveils potential KRAS inhibitors. Nat. Biotechnol..

[29] McArdle S., Endo S., Aspuru-Guzik A., Benjamin S., Yuan X. (2020). Quantum computational chemistry. Rev. Mod. Phys..

[30] Peruzzo A., McClean J., Shadbolt P., Yung M.H., Zhou X.Q., Love P.J. (2014). A variational eigenvalue solver on a photonic quantum processor. Nat. Commun..

[31] Reiher M., Wiebe N., Svore K.M., Wecker D., Troyer M. (2017). Elucidating reaction mechanisms on quantum computers. Proc. Natl. Acad. Sci. USA.

[32] Aspuru-Guzik A., Dutoi A.D., Love P.J., Head-Gordon M. (2005). Chemistry: Simulated quantum computation of molecular energies. Science.

[33] Tuckerman M.E. (2002). Ab initio molecular dynamics: Basic concepts, current trends and novel applications. J. Phys. Condens. Matter.

[34] Benedetti M., García-Pintos D., Perdomo O., Leyton-Ortega V., Nam Y., Perdomo-Ortiz A. (2019). A generative modeling approach for benchmarking and training shallow quantum circuits. npj Quantum Inf..

[35] Preskill J. (2018). Quantum Computing in the NISQ era and beyond. Quantum.

[36] Rembold C.M. (1998). Number needed to screen: Development of a statistic for disease screening. Br. Med. J..

[37] Leeflang M.M.G., Bossuyt P.M.M., Irwig L. (2009). Diagnostic test accuracy may vary with prevalence: Implications for evidence-based diagnosis. J. Clin. Epidemiol..

[38] Brisinda D., Fenici P., Fenici R. (2023). Clinical magnetocardiography: The unshielded bet—Past, present, and future. Front. Cardiovasc. Med..

[39] Strand S., Lutter W., Strasburger J.F., Shah V., Baffa O., Wakai R.T. (2019). Low-Cost Fetal Magnetocardiography: A Comparison of Superconducting Quantum Interference Device and Optically Pumped Magnetometers. J. Am. Heart Assoc..

[40] Barratt A., Irwig L., Glasziou P., Cumming R.G., Raffle A., Hicks N., Gray J.M., Guyatt G.H. (1999). Users’ Guides to the Medical Literature XVII. How to Use Guidelines and Recommendations About Screening. JAMA.

[41] Mushlin A.I., Ruchlin H.S., Callahan M.A. (2001). Cost-effectiveness of diagnostic tests. Lancet.

[42] Schuld M., Sinayskiy I., Petruccione F. (2014). The quest for a Quantum Neural Network. Quantum Inf. Process..

[43] Eze-Nliam C.M., Zhang Z., Weiss S.A., Weintraub W.S. (2014). Cost-effectiveness assessment of cardiac interventions: Determining a socially acceptable cost threshold. Interv. Cardiol..

[44] Topol E.J. (2019). High-performance medicine: The convergence of human and artificial intelligence. Nat. Med..

[45] Rajkomar A., Dean J., Kohane I. (2019). Machine Learning in Medicine. N. Engl. J. Med..

[46] Obermeyer Z., Emanuel E.J. (2016). Predicting the Future—Big Data, Machine Learning, and Clinical Medicine. N. Engl. J. Med..

[47] Chen J.H., Asch S.M. (2017). Machine Learning and Prediction in Medicine—Beyond the Peak of Inflated Expectations. N. Engl. J. Med..

[48] Awschalom D.D., Hanson R., Wrachtrup J., Zhou B.B. (2018). Quantum technologies with optically interfaced solid-state spins. Nat. Photonics.

[49] Hood L., Friend S.H. (2011). Predictive, personalized, preventive, participatory (P4) cancer medicine. Nat. Rev. Clin. Oncol..

[50] Ismail M.I., Qaswal A.B., Ali M.A.B., Hamdan A., Alghrabli A., Harb M., Ibrahim D., Al-Jbour M.N., Almobaiden I., Alrowwad K. (2023). Quantum mechanical aspects of cardiac arrhythmias: A mathematical model and pathophysiological implications. AIMS Biophys..

[51] Ababneh O., Qaswal A.B., Alelaumi A., Khreesha L., Almomani M., Khrais M., Khrais O., Suleihat A., Mutleq S., Al-olaimat Y. (2021). Proton Quantum Tunneling: Influence and Relevance to Acidosis-Induced Cardiac Arrhythmias/Cardiac Arrest. Pathophysiology.

[52] Hille B. (1978). Ionic Channels in Excitable Membranes Current Problems and Biophysical Approaches. Biophys. J..

[53] Bezanilla F. (2008). How membrane proteins sense voltage. Nat. Rev. Mol. Cell Biol..

[54] Engel G.S., Calhoun T.R., Read E.L., Ahn T.K., Mančal T., Cheng Y.C., Blankenship R.E., Fleming G.R. (2007). Evidence for wavelike energy transfer through quantum coherence in photosynthetic systems. Nature.

[55] Ritz T., Adem S., Schulten K. (2000). A Model for Photoreceptor-Based Magnetoreception in Birds. Biophys. J..

