# Correction: Tomala, M.; Kłaczyński, M. Quantum Cardiovascular Medicine: From Hype to Hope—A Critical Review of Real-World Applications. *J. Clin. Med.* 2025, *14*, 6029

**DOI:** 10.3390/jcm15197730

**Published:** 2026-10-08

**Authors:** Marek Tomala, Maciej Kłaczyński

**Affiliations:** 1 Center for Invasive Cardiology, Electrotherapy, and Angiology, 33-300 Nowy Sącz, Poland; 2 Clinical Research Center Intercard, 30-514 Krakow, Poland; 3 Department of Mechanics and Vibroacoustics, Faculty of Mechanical Engineering and Robotics, AGH University of Krakow, Al. Mickiewicza 30, 30-059 Krakow, Poland; maciej.klaczynski@agh.edu.pl

## 1. Correction of Figures 2–4

### 1.1. Figure 2 (SQUID Magnetometer)

In the original publication [1], Figure 2 was created using automated graphical tools and had the following errors. In the lower right corner of the image, the label “magnetic loup” should have read “magnetic loop”.

The interior of the superconducting loop was filled with wavy lines called “Quantum interference patterns,” but these lines have no physical significance related to SQUID operation. Quantum interference in a DC SQUID produces a periodic voltage output, V = f(Φ/Φ_0_) rather than showing a spatial representation of the loop’s interior. The image also lacked standard quantitative annotations; it did not show the screening current flowing around the loop (I), the output voltage (V = f(Φ/Φ_0_)), or the flux quantum (Φ_0_ = h/2e ≈ 2.07 × 10^−15^ Wb). The two Josephson junctions were illustrated as 3D rectangular blocks, with only one labeled, instead of using the standard × notation with separate labels for each junction, J_1_ and J_2_. The magnetic flux lines were depicted radiating from the device in the wrong direction, suggesting that the SQUID produces magnetic fields rather than detecting them.

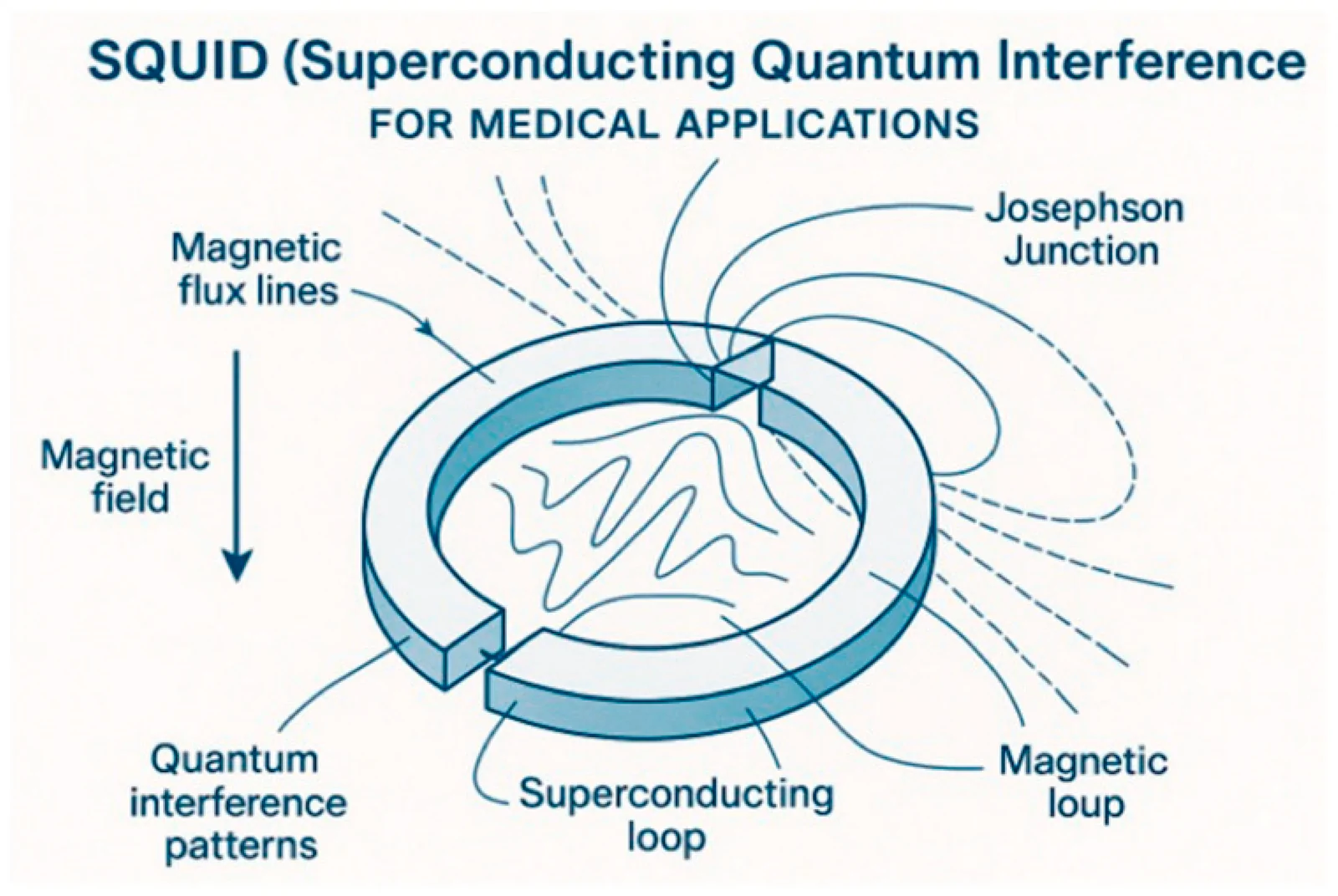



All of the issues mentioned above were addressed by revising Figure 2. The superconducting loop is shown on the left panel of the figure, containing two Josephson junctions labeled J_1_ and J_2_, using the standard × notation for each. The circulating screening current (I) and the output voltage V = f(Φ/Φ_0_) are explicitly indicated and shown on the right side of the panel. The right side depicts a typical periodic relationship between voltage and flux, including the working point (max dV/dΦ) and the voltage swing ΔV. A summary of several important physical parameters—such as Φ_0_ = h/2e ≈ 2.07 × 10^−15^ Wb, magnetic sensitivity ~1 fT/√Hz, and an operating temperature below 4.2 K—can be found in the accompanying Table.

### 1.2. Figure 3 (Nitrogen-Vacancy Center in Diamond)

Figure 3 was generated using automated graphical tools and contained the following errors.

The most fundamental error concerned the NV defect configuration. The original figure depicted one nitrogen atom flanked by two vacancies—one above and one below—whereas the correct structure of an NV center consists of exactly one substitutional nitrogen atom adjacent to a single carbon vacancy, with the N–V pair aligned along the ⟨111⟩ crystallographic axis. The ⟨111⟩ axis was not indicated anywhere in the original figure.

The crystal lattice shown in the original figure did not accurately represent the diamond cubic structure. The arrangement of carbon atoms appeared geometrically hexagonal rather than reflecting the face-centered cubic Fd$\bar{3}$m space group of diamond, which is defined by tetrahedral sp^3^ coordination with characteristic bond angles of 109.5°.

The right panel of the original figure contained no physically meaningful energy level information. It showed only generic labels—“Spin States,” “Quantum Coherence,” and “Room Temperature Operation”—without any representation of the actual electronic structure of the NV^−^ center. Absent from the original were: the spin-triplet ground state (^3^A_2_) with its m_s_ = 0 and m_s_ = ±1 sublevels, the zero-field splitting (D = 2.87 GHz), the excited triplet state (^3^E), the intermediate singlet states (^1^A_1_ and ^1^E), the intersystem crossing (ISC) pathways responsible for spin-dependent fluorescence, and the optical excitation and emission wavelengths (532 nm and 637–800 nm, respectively).

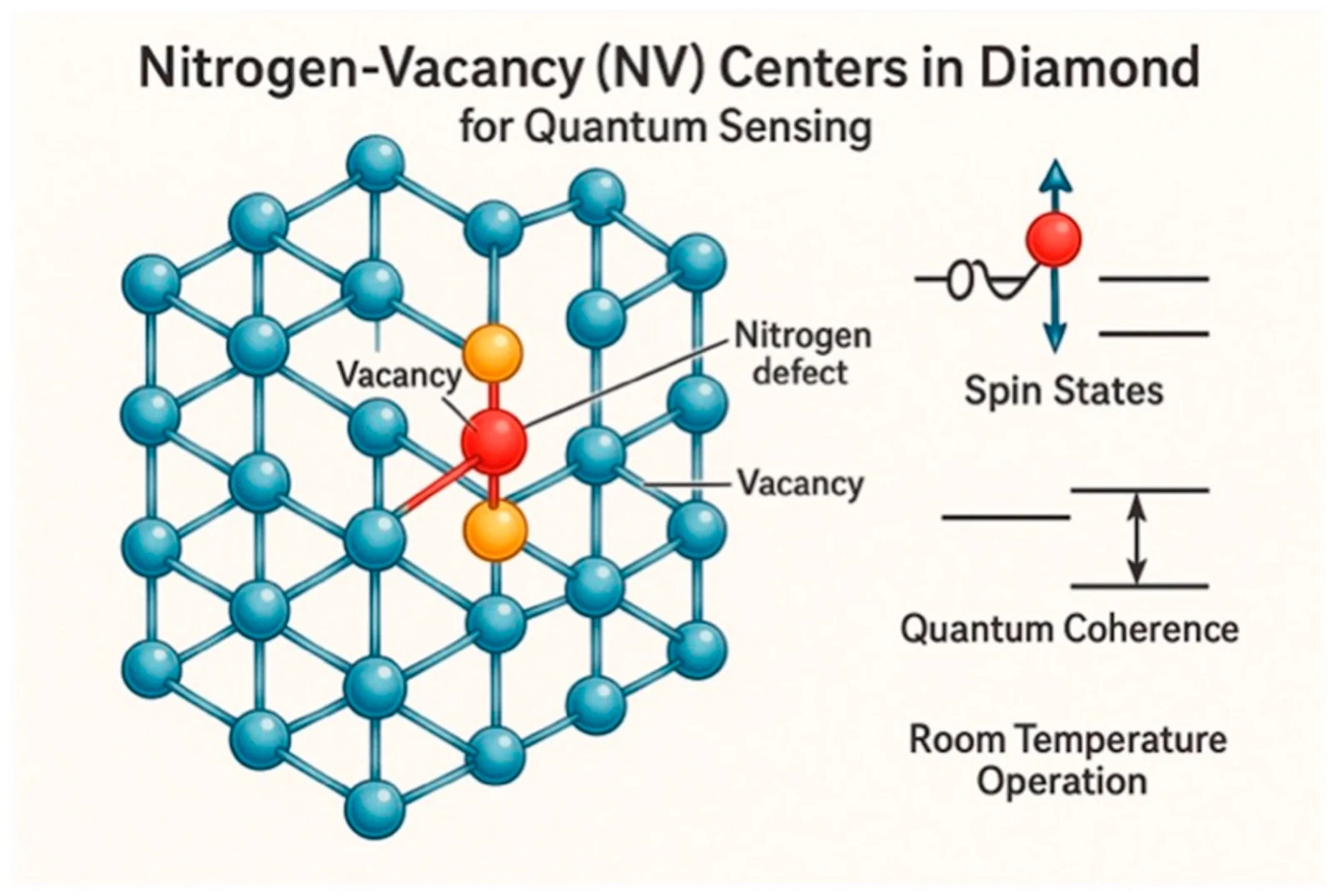



The corrected Figure 3 addresses each of these points. The left panel shows the diamond Fd$\bar{3}$m lattice with correct tetrahedral geometry, a bond length of 1.54 Å, and bond angles of 109.5°, with one substitutional nitrogen atom (N) adjacent to one vacancy (V) along the explicitly labeled ⟨111⟩ axis. The right panel presents the complete NV^−^ energy level structure, including the spin-triplet ground-state sublevels, zero-field splitting D = 2.87 GHz, the excited state ^3^E, the singlet states ^1^A_1_ and ^1^E with ISC pathways, and optical transitions at 532 nm excitation and 637–800 nm fluorescence. Key performance parameters—operating temperature 300 K and spin coherence T_2_ of 1–10 ms—are summarized in the accompanying Table.

### 1.3. Figure 4 (Optically Pumped Magnetometer)

Figure 4 was created using automated graphical tools and contained several errors. The most significant mistake was in the energy-level diagram. The original figure showed two anonymous energy levels, labeled E_1_ and E_2_, without reference to the alkali atom’s hyperfine structure or the quantum numbers governing OPM operation. An OPM in the SERF regime detects the Zeeman splitting of the ground-state hyperfine sublevels (m_F_) due to an external magnetic field B, with an energy shift given by ΔE = g_F_·μ_B_·B. None of this was depicted in the original figure. Related to this was a fundamental misrepresentation of Larmor precession. In the original diagram, precession was shown as a spiral arrow connecting E_1_ to E_2_, implying a transition between energy levels. In reality, Larmor precession is the coherent rotation of the spin-polarization vector within the ground-state sublevels, not a transition from one energy level to another, and its frequency, ω_L_ = γ·B, is directly proportional to the external magnetic field strength. This rotational motion is what the probe beam measures, but the misrepresentation obscures the device’s core sensing mechanism. The original figure also repeated the term “optical detection” twice, once next to an upward arrow and again next to a downward arrow, without clarifying whether it referred to the pump or the probe beams. Therefore, the two-beam operation of the OPM—where a circularly polarized pump beam induces spin polarization and a linearly polarized probe beam measures the resulting precession by detecting optical rotation—could not be understood from the figure alone. Lastly, the magnetic field on the right side was depicted as a series of curved, radially propagating lines, representing electromagnetic radiation rather than the static magnetic field of the target being measured by OPMs.

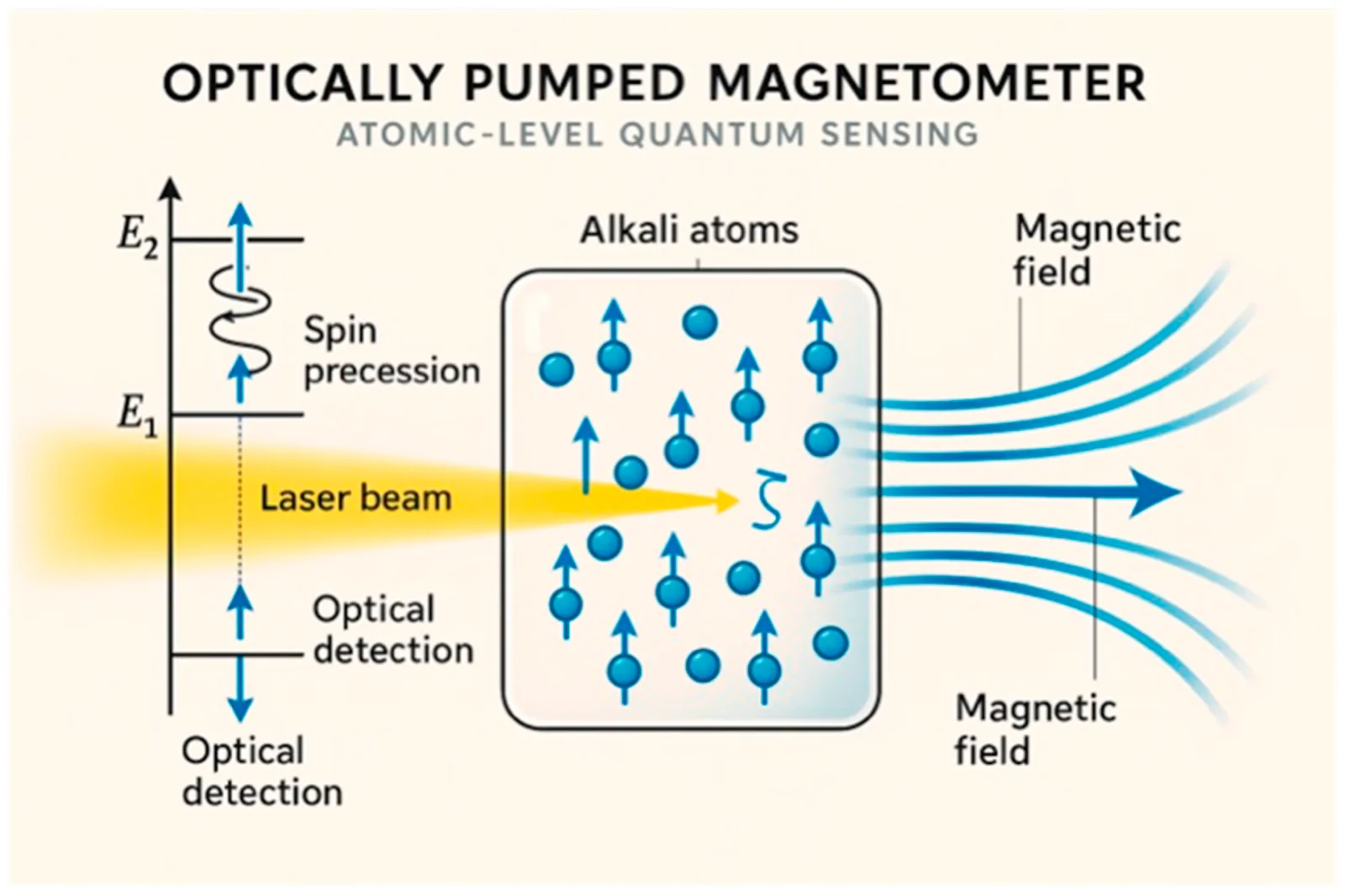



The corrected version of Figure 4 addresses all these issues. It shows, on the left, the entire OPM system: the alkali-vapor cell containing ^87^Rb atoms, the circularly polarized pump beam (795 nm, σ^+^), the linearly polarized probe beam (780 nm) measuring optical rotation signals (α, β) corresponding to the magnetic field strength, and the photodetector. The magnetic field from the target is shown as a directional quasi-static vector. The right side features the correct Zeeman-splitting diagram for the hyperfine structure of the ^87^Rb ground state, including the sublevels of F = 2 (m_F_ = +2, +1, 0, −1, −2) and F = 1 (m_F_ = +1, 0, −1). Additionally, the energy shift equation ΔE = g_F_·μ_B_·B and the Larmor frequency expression (ω_L_ = γ·B) are included. A summary Table provides key performance characteristics of the SERF regime, including sensitivity (1–10 fT/√Hz), bandwidth (DC–100 Hz), and operating temperature (approximately 150 °C for the cell and room temperature for the device).

## 2. Disclosure Regarding the Use of Automated Graphical Tools

The authors acknowledge that the original Figures 2–4 were generated using automated graphical tools. This was not disclosed in the original manuscript, which was an oversight inconsistent with MDPI’s editorial policies on AI-assisted content generation. The authors confirm that automated tools were solely used to create the three illustrative figures and did not affect the textual content, data analysis, or conclusions of the review. The corrected figures provided herein were prepared using standard vector graphics software without the aid of automated generation tools.

The authors used Grammarly (Grammarly Inc.) to correct the manuscript’s grammar and language. The authors reviewed and edited the content as needed and take full responsibility for the publication’s content.

## 3. Impact on Scientific Content

The authors wish to emphasize that the errors in the figures were limited to the illustrative schematics (Figures 2–4), which were intended as conceptual diagrams in this narrative review. The textual descriptions of SQUID, NV center, and OPM technologies in the manuscript body remain scientifically accurate and are supported by the cited literature. Figure 1 (PRISMA flow diagram) was not generated by automated tools and is unaffected by this correction. No changes to the textual content, methodology, results, or conclusions of the review are required.

The authors state that the scientific conclusions are unaffected. This correction was approved by the Academic Editor. The original publication has also been updated.

## Figures and Tables

**Figure 2:**
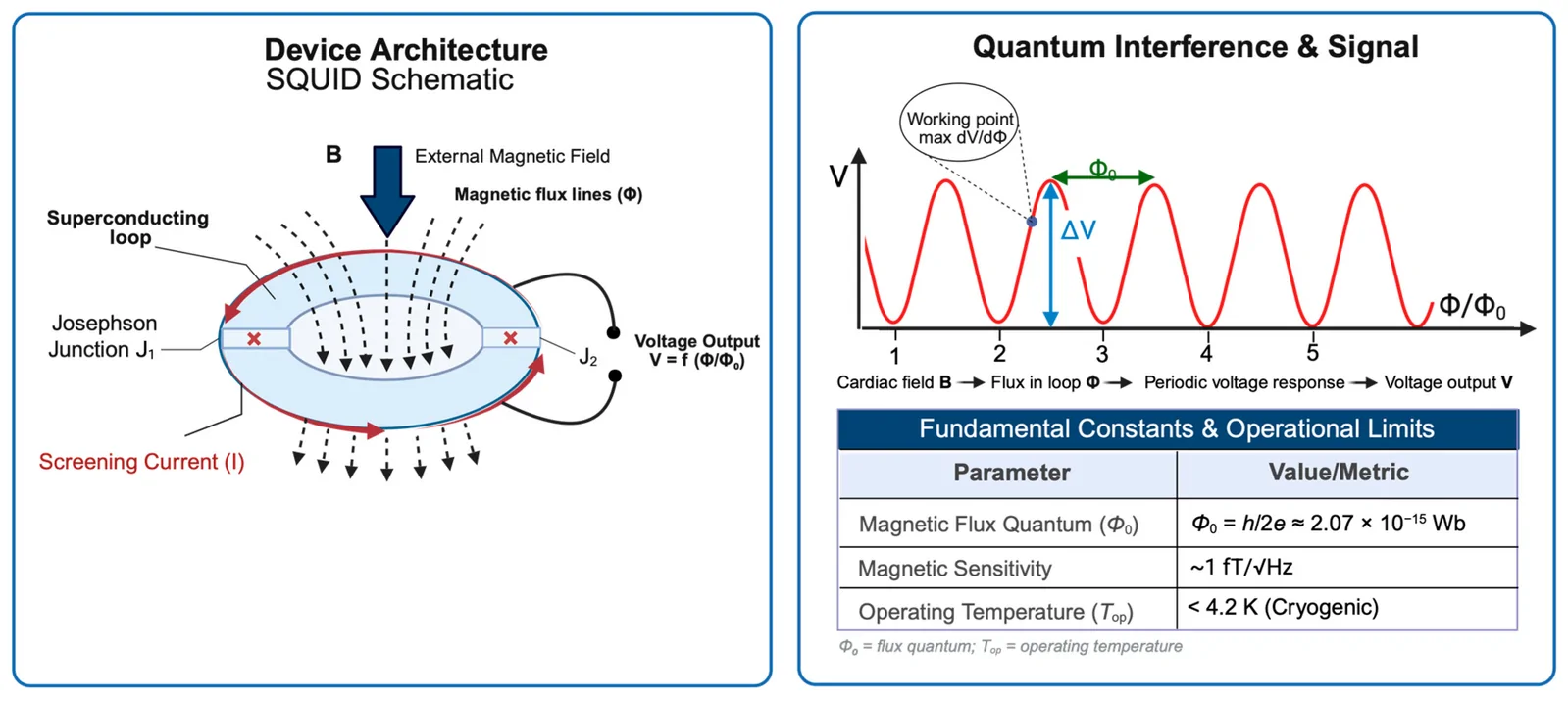
The structure of a DC SQUID (Superconducting Quantum Interference Device) magnetometer for biomedical applications and the quantum interference signal it produces. The **left** panel shows the superconducting loop that contains two Josephson junctions (J_1_ and J_2_, denoted by ×), a screening current I flowing around the loop, and a voltage V = f(Φ/Φ_0_) across the junction pair, periodically modulated by the externally applied magnetic field B, which creates magnetic flux lines (Φ) through the loop. The **right** panel shows the voltage–flux relationship (V vs. Φ/Φ_0_) for the SQUID, including the optimal working point (maximum dV/dΦ) and the voltage swing (ΔV). The relevant device parameters are shown in the Table below: Φ_0_ = h/2e ≈ 2.07 × 10^−15^ Wb; magnetic sensitivity ~1 fT/√Hz (fT: femtotesla); and operating temperature T_op_ < 4.2 K (cryogenic).

**Figure 3:**
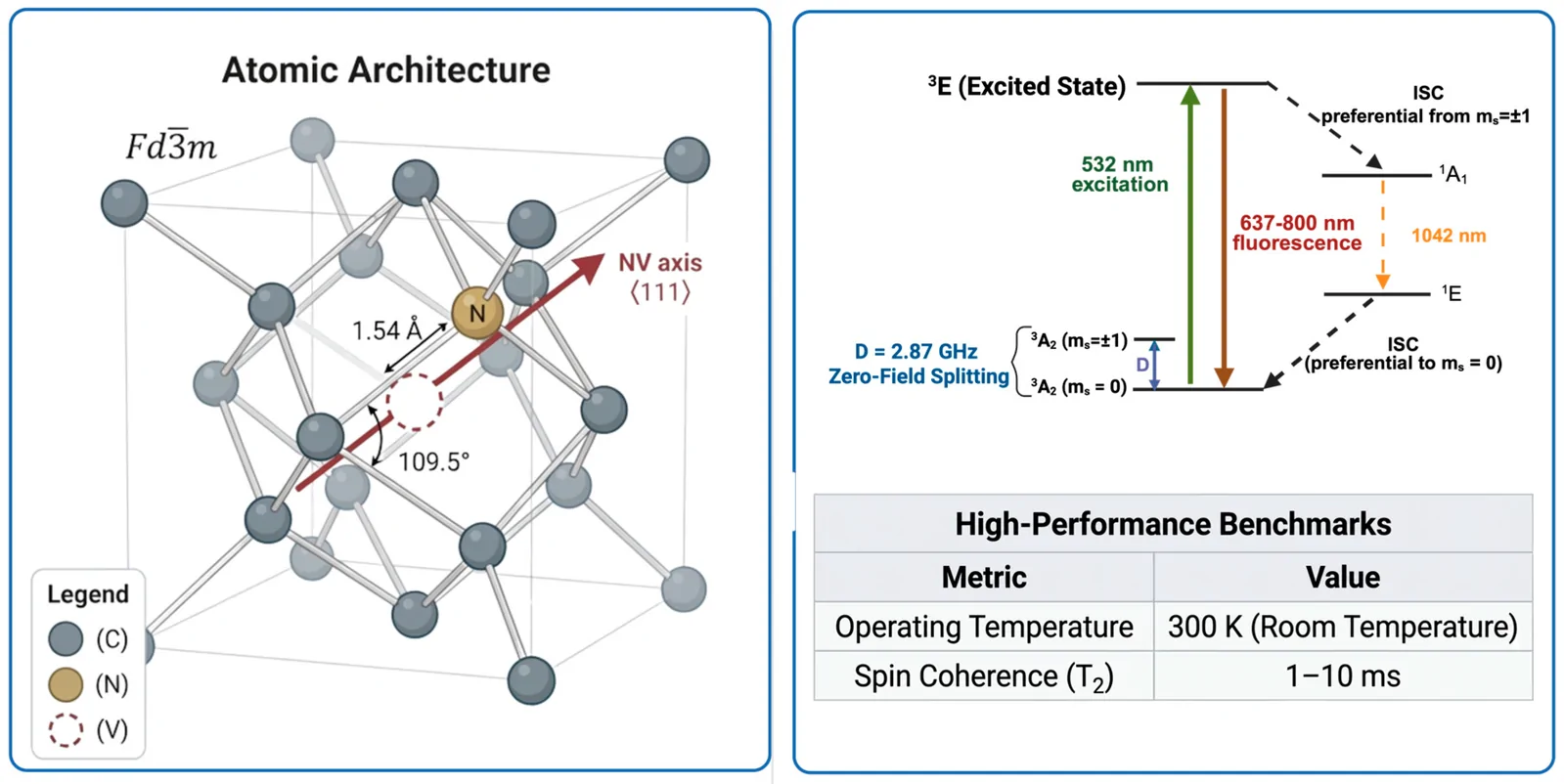
Atomic structure of nitrogen-vacancy center in diamond and energy level transitions for quantum sensing. **Left** panel: nitrogen-vacancy (NV^−^) defect in the diamond cubic lattice (Fd$\bar{3}$m). A substitutional nitrogen atom (N, gold) is adjacent to a single carbon vacancy (V, dashed circle) along the ⟨111⟩ crystallographic direction. Carbon atoms (C, gray) are arranged in a tetrahedral lattice, forming bonds 1.54 Å in length and 109.5° in angle. **Right** panel: optical spin-polarization and readout cycle of the NV^−^ center at zero magnetic field: light at 532 nm excites the system from the Spin-Triplet Ground State ^3^A_2_ to the Excited State ^3^E; fluorescence emitted at wavelengths between 637 and 800 nm; zero-field splitting (D = 2.87 GHz) of the ground state creates two energy levels: m_s_ = 0 and the doubly degenerate m_s_ = ±1; population of the m_s_ = 0 substate is preferential due to Intersystem Crossing (ISC) pathways through the singlet states ^1^A_1_ and ^1^E, which are coupled by the 1042 nm infrared transition; enables optical readout of spin-state. Relevant Performance Metrics for NV^−^ are listed in the Table below: operating temperature = 300 K (room temperature); coherence time of the Spin State (T_2_): 1–10 ms.

**Figure 4:**
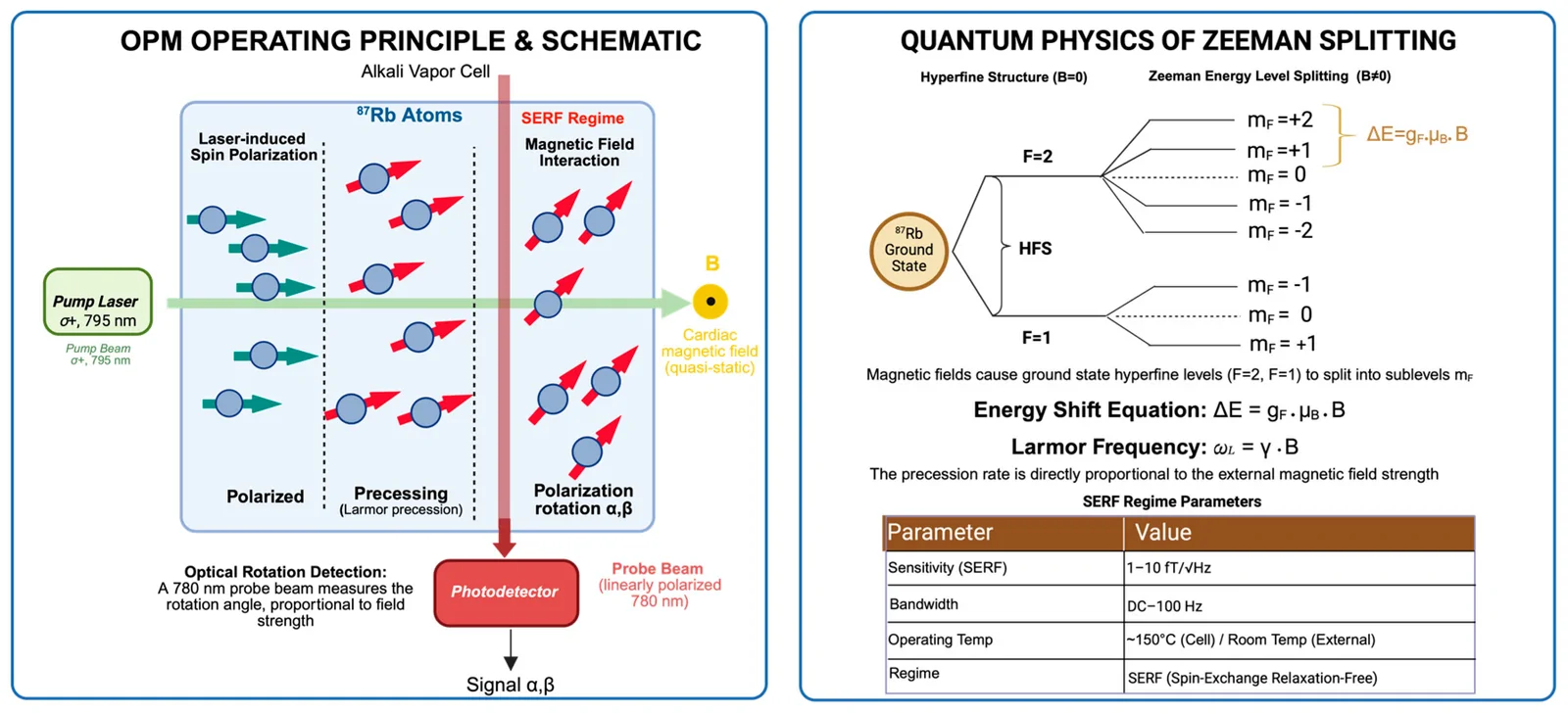
The Optically Pumped Magnetometer (OPM) operating principle and quantum physics of Zeeman splitting in the SERF regime. **Left** panel: a circularly polarized pump beam (σ^+^, 795 nm) generates spin polarization of ^87^Rb atoms contained within the alkali vapor cell; a linearly polarized probe beam (780 nm) crosses the cell perpendicular to the pump beam, through the polarization-rotation region, thereby generating an optical rotation signal (α, β) measured at the photodetector that is proportional to the quasi-static external cardiac magnetic field (B); the three labeled regions—polarized, precessing (Larmor precession), and polarization rotation—mark successive stages in the evolution of the spin ensemble along the pump direction. **Right** panel: an externally applied magnetic field (B) splits the hyperfine states of the ^87^Rb ground state due to the Zeeman effect, resulting in energy shifts in the hyperfine states (ΔE = g_F_·μ_B_·B) with the Larmor frequency ω_L_ = γ·B being directly proportional to the field strength. This represents the basic principle of the OPM measurement. Relevant Performance Metrics for the SERF regime are listed in the Table below: sensitivity = 1–10 fT/√Hz (fT: femtotesla); bandwidth = DC–100 Hz; operating temperature ≈ 150 °C (cell)/room temperature (external); regime = SERF (Spin-Exchange Relaxation-Free). Solid colored arrows indicate the optical beams (green: σ^+^ pump, 795 nm; dark red: 780 nm probe); the external magnetic field B is shown as a yellow circled dot, showing that it is directed out of the plane of the figure, perpendicular to both beams. The small arrows within the vapor cell represent atomic spin orientation, aligned in the polarized region and tilted during Larmor precession. In the right panel, horizontal lines denote the hyperfine/Zeeman energy sublevels m_F_; the m_F_ = 0 sublevel is drawn as a dashed line because it is not shifted to first order in B, and the slanted lines are guides connecting each hyperfine manifold F to its shifted sublevels. The m_F_ ordering is inverted between the F = 2 and F = 1 manifolds because the hyperfine Landé factor g_F_ has opposite signs for F = 2 and F = 1.
